# Evolution of birth-cohort patterns in lifestyle-associated cancer mortality across high-income countries

**DOI:** 10.1093/ije/dyag198

**Published:** 2026-10-06

**Authors:** Octavio N Bramajo, Leah R Abrams, Neil K Mehta

**Affiliations:** Institute for Evolutionary Medicine, University of Zürich, Zürich, Switzerland; Aarhus Institute for Advanced Studies (AIAS), Aarhus University, Aarhus C, Denmark; Department of Community Health, Tufts University, Boston, MA, United States; Department of Epidemiology, University of Texas Medical Branch, Galveston, TX, United States

**Keywords:** cancer epidemiology, demography, public health, visual methods

## Abstract

**Background:**

Cancer mortality levels have declined across high-income countries, but progress has been uneven by cancer type. Examining birth-cohort patterns of lifestyle-related cancer mortality in an international context can shed light on how shared and differential global lifestyle transitions manifest differently across national contexts.

**Methods:**

We used harmonized cause-of-death data from the Human Cause of Death series of the Human Mortality Database for Canada, Czechia, England & Wales, France, Germany, Japan, Spain, and the USA from 2000 to 2020. We examined mortality from lung and larynx, colorectal, stomach, and liver cancer for ages 30–84 years by using rates of mortality improvement displayed on Lexis surfaces. Single-age death rates were estimated from grouped data by using penalized composite link models. Two-factor Poisson models and sex-stratified surfaces served as sensitivity analyses.

**Results:**

All eight countries showed declining age-standardized cancer mortality, but with diverging trends by cause and birth cohorts. Lung and larynx cancer declined broadly, with mortality patterns reflecting cohort cigarette-smoking histories. Colorectal cancer mortality increased among cohorts born after 1960 in the USA, Canada, and England & Wales, with a similar but delayed pattern in France and Germany. Liver cancer mortality was higher for cohorts born in 1945–55 than for earlier and later cohorts in several countries, with a re-emerging adverse pattern among cohorts born in around 1970–80.

**Conclusion:**

Birth-cohort patterns in lifestyle-associated cancer mortality differ across national contexts. Worsening colorectal and liver cancer trends among younger cohorts call for increased prevention and screening strategies.

Key Messages

- Despite overall improvements in cancer mortality levels, prior studies have highlighted adverse trends in certain lifestyle-related cancers. Prior studies have not conducted a detailed birth-cohort analysis across high-income countries.
- Colorectal cancer mortality is rising among cohorts born after 1960 in the USA, Canada, and England & Wales, with a similar but delayed pattern in Germany and France; liver cancer shows a re-emerging adverse signal among cohorts born in around 1970–80.
- Cross-national cancer trajectories tend to be similar, with the USA, Canada, and England & Wales leading adverse trends and France and Germany following approximately one decade behind.
- Findings indicate lifestyle environments as a primary driver of cohort trends and offer a forewarning for prevention policy.

## Introduction

Declines in death rates from cancer represent one of the most significant public health achievements of the past several decades [1]. The decline is attributable to several factors, including the recession of the smoking epidemic, better early detection, and treatment innovations. However, progress has not been uniform across time, cancer type, or population [2].

While most analyses on cancer trends focus on changes by calendar year, attention has also been given to patterns by birth cohorts. The cohort perspective recognizes that individuals born in the same time period (i.e. generations) share similar environmental exposures and health behaviours as they age that can distinctly influence disease risk throughout their lifetime [3]. The differing risk profiles and lifestyle patterns across generations can be associated with specific forms of cancer [4].

Cohort patterns in cancer mortality are most prominent for lung and larynx cancers—diseases that are largely driven by cigarette-smoking behaviour [5–8]. There is evidence that, when tobacco-control policies resulted in a reduction in the prevalence of smoking among men in several high-income countries, there was a decline in lung cancer incidence and mortality ∼20–30 years later [2]. While the impact of cigarette smoking on cancer risk has been documented since the 1950s, in some regions, particularly in Europe, lung cancer mortality is still rising [9–11].

Colorectal cancer is also driven by behavioural patterns that may be patterned by birth cohort, including the effects of diet, physical activity, and obesity [12, 13]. The prevalence of colorectal cancer has been increasing in recent years, especially in younger birth cohorts and with younger age of onset [14–16]. In the USA and Canada, mortality rates from colorectal cancer are expected to increase by 25% by 2035 [17].

Liver cancer is strongly associated with alcohol consumption [18], hepatitis B and C virus infection, and non-alcoholic steatohepatitis. After a steady decline in 1990–2010, liver cancer mortality rose in 2010–19 [19], suggesting that, like colorectal cancer, cohort-based changes in lifestyles may be patterning recent trends.

Stomach cancer is predominantly driven by *Helicobacter pylori* infection and there is evidence that improved sanitation and living standards have decreased the rates of gastric cancer incidence and mortality over time [20]. However, the disease is also influenced by diet, alcohol, and sodium intake, with adverse cohort trends documented in some American and European populations [21, 22].

While cohort analysis has been applied to cancer research [4], comprehensive international comparisons using this framework remain limited. Some existing studies rely on age–period–cohort models—a statistical framework that presents a fundamental identification problem: because age, period, and cohort are perfectly collinear, no unique solution exists without the imposition of arbitrary constraints that often cannot be justified [23]. Graphical representations of trends across age and year can provide a clearer understanding of birth-cohort patterns in cancer mortality [24–26].

This study uses a birth-cohort perspective that does not rely on the assumptions of age–period–cohort models, but rather relies on a detailed visualization of mortality trends in a cross-national framework (Canada, Czechia, England & Wales, France, Germany, Japan, Spain, and USA). Focusing on lung and larynx, colorectal, stomach, and liver cancers, which each represent different degrees of lifestyle influence, we examine whether countries have shown similar generational patterns of risk over the past two decades and explore how shared global lifestyle transitions manifest differently across national contexts.

## Methods

### Data sources

Mortality data by birth year and cause were obtained from the Human Cause of Death Data Series (HCD) [27], a component of the Human Mortality Database (HMD) project [28]. We selected Canada, Czechia, England & Wales, France, Germany, Japan, Spain, and the USA because they have available and harmonized cause-of-death data for the period when ICD-10 classifications were adopted for 2000–20, making cause-specific death rates comparable across countries without additional considerations. The sole exception is England & Wales, whose HCD series began in 2001. While 2020 was an exceptional year for all-cause mortality, the longer-running trends for cancer mortality continued in the analysed countries [11].

In addition to total cancer mortality, we examined specific cancer subgroups based on their mortality burden and lifestyle associations. We selected cancers with strong lifestyle components, including lung and larynx cancer and colorectal cancer, as well as those with partial lifestyle associations, such as stomach cancer and liver cancer. All remaining cancers were grouped as “other cancers” and are presented in the Supplementary Material. Sex-specific reproductive cancers (breast, prostate, cervix, uterus, and ovary) were not considered individually, as they are driven mainly by hormonal and reproductive rather than behavioural factors; they are included within the “other cancers.” The complete list of ICD-10 codes and corresponding HCD codes is provided in the Supplementary Material.

We analysed deaths from these cancers for ages of 30–84 years. Sex-specific analyses are available in the Supplementary Material. Ill-defined deaths were redistributed in the estimates according to HCD protocols. As this study was a secondary analysis of existing de-identified publicly available data, Institutional Review Board approval was not required under 45 Code of Federal Regulations §46.101(b)(4). The study followed the Strengthening the Reporting of Observational Studies in Epidemiology (STROBE) reporting guideline [29].

### Statistical analysis

To explore secular changes, we first calculated age-standardized mortality rates by cause and country for the period 2000–20. We used the 2010 US age structure for both sexes as the standard population—an actual national age distribution at the midpoint of the study period, which facilitates benchmarking against other studies. This standard affects mortality levels, but has no influence on comparisons of mortality change across cohorts, countries, and time.

To illustrate mortality change by birth cohort, we used Lexis surfaces—a visual approach for plotting mortality rates or their changes on a 2D grid of age (*y*-axis) and calendar year (*x*-axis). Cohort patterns appear as diagonal bands running from the lower-left to upper-right of the diagram. In cancer epidemiology, Lexis surfaces have recently been applied to mortality trends in Hungary [25] and the USA [24].

We plotted the “change” in mortality—what Rau and colleagues term “rates of mortality improvement” or “ROMI” (*∂*) [26]. For each age group, *ρ* is the annual change in the logarithm of the mortality rate (mx), *ρ* at age *a* and time *t*, which approximates the proportionate annual change and is reported as a percentage, as shown in Equation (1):


(1) $$\partial_{a,t}\approx \log\;{\mathrm{mx}}_{a,t+1}\;(a,t+1)-\log\;{\mathrm{mx}}_{a,t}\;$$

A positive value of 1% signifies a 1% increase in mortality for a given age group compared with that age group in the previous year, while −1% signifies a 1% decrease. Contour lines at 0% differentiate increasing and decreasing trends in mortality. To ensure that the visualizations were comprehensible and comparable, we constrained extreme values (which were rare and limited to the extreme upper ages) to ±15%.

HCD-series data are provided in 5-year age groups. ROMI surfaces perform better with smoothed single-age data [26]. To obtain death rates by single ages, we applied a 2D penalized composite link model, available in the ungroup package in R [30], specifically designed for handling grouped data using population exposure as an offset. This method requires only the assumptions that age-specific death counts are Poisson-distributed and that the mortality distribution is smooth. Sex-stratified Lexis surfaces by cause are available in the Supplementary Figures A2–A13.

We conducted two sensitivity analyses. First, we replicated the analysis without smoothing by using 5-year age groups in tile plots (Supplementary Figures A14–A20). Second, we estimated a series of two-factor age–period and age–cohort Poisson log-linear models to examine whether the observed patterns reflected robust changes. All models were run separately by cause and country; the results are presented in Supplementary Figures A21–A35.

## Results

The age-standardized death rates by country and year, separately by cancer type, are presented in Fig. 1. Between 2000 and 2020 (2001–20 for England & Wales), age-standardized all-cancer mortality declined in all eight countries. Relative declines ranged from 29% in the USA (from 358.2 to 255.5 deaths per 100 000) to 17% in Japan (347.6 to 287.1); absolute rates by country and cancer type for 2000, 2010, and 2020 are shown in Table 1.

**Figure 1:**
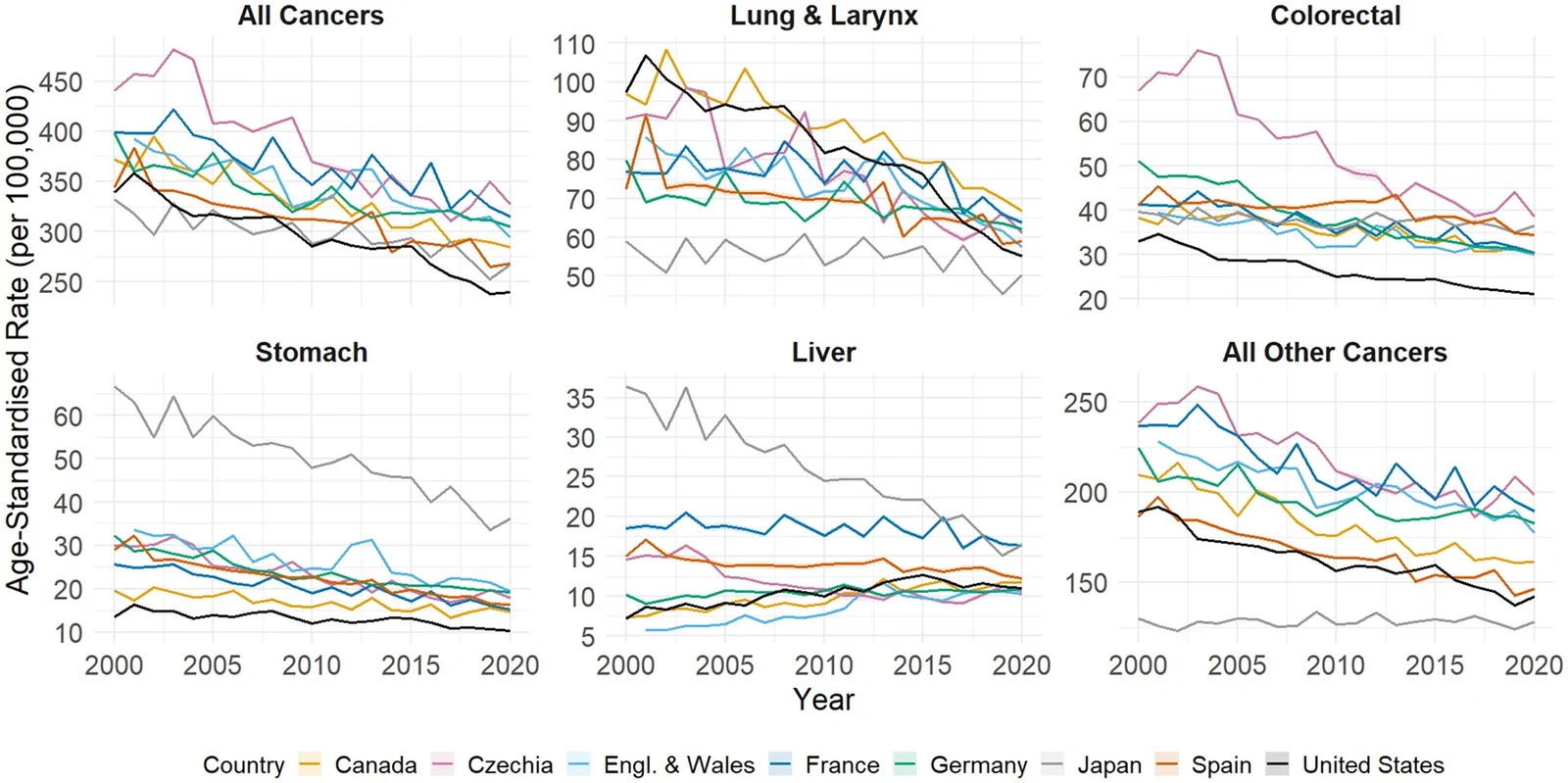
Age-standardized rates of mortality (per 100 000) across eight countries between 2000 and 2020 for all cancers, with 95% Confidence Intervals. Data from HCD.

**Table 1:** Age-standardized rates of mortality (per 100 000) across eight countries for baseline year (2000; 2001 in England & Wales), 2010, and 2020 by cancer type. Data from HCD.

Country	Type	Baseline (2000 or 2001)	2010	2020
Canada	All cancers	391.8	343.9	303.7
Czechia	All cancers	477.8	396.1	344.9
England & Wales	All cancers	398.6	359.4	328.5
France	All cancers	413.8	363.2	334.1
Germany	All cancers	391.7	344.6	321.6
Japan	All cancers	347.6	316.4	287.1
Spain	All cancers	358.9	326.5	283.5
USA	All cancers	358.2	307.8	255.5
Canada	Lung and larynx	101.6	93.0	71.0
Czechia	Lung and larynx	98.6	80.5	63.7
England & Wales	Lung and larynx	84.2	78.4	63.5
France	Lung and larynx	77.6	75.5	66.3
Germany	Lung and larynx	73.7	70.2	64.4
Japan	Lung and larynx	61.8	60.1	53.9
Spain	Lung and larynx	74.0	71.7	61.2
USA	Lung and larynx	106.2	88.5	59.3
Canada	Colorectal	41.3	36.9	32.3
Czechia	Colorectal	74.6	54.6	41.5
England & Wales	Colorectal	40.5	35.7	33.9
France	Colorectal	43.6	37.1	32.7
Germany	Colorectal	52.2	39.0	32.4
Japan	Colorectal	41.8	39.3	39.1
Spain	Colorectal	43.5	44.3	36.9
USA	Colorectal	35.6	27.1	22.5
Canada	Stomach	20.8	17.4	15.5
Czechia	Stomach	33.0	24.6	19.1
England & Wales	Stomach	33.6	28.0	22.8
France	Stomach	26.6	19.8	15.8
Germany	Stomach	31.5	23.7	20.0
Japan	Stomach	69.9	53.9	38.7
Spain	Stomach	30.1	23.7	17.2
USA	Stomach	16.1	13.9	11.6
Canada	Liver	7.8	9.9	12.4
Czechia	Liver	15.8	11.9	10.2
England & Wales	Liver	5.8	8.6	11.8
France	Liver	19.0	18.4	17.2
Germany	Liver	9.8	11.2	11.4
Japan	Liver	37.5	27.7	17.7
Spain	Liver	15.6	14.6	12.9
USA	Liver	8.4	11.0	12.1
Canada	All other cancers	220.2	186.8	172.5
Czechia	All other cancers	255.8	224.4	210.4
England & Wales	All other cancers	234.4	208.8	196.5
France	All other cancers	247.0	212.5	202.1
Germany	All other cancers	224.5	200.5	193.3
Japan	All other cancers	136.5	135.4	137.7
Spain	All other cancers	195.6	172.3	155.2
USA	All other cancers	191.8	167.2	150.1

The decline in overall cancer mortality was driven largely by reductions in lung and larynx, colorectal, and stomach cancers, although colorectal cancer has shown a slower pace of decline across many countries since 2005, with stagnation in some cases. In contrast, liver cancer mortality increased in several countries or stagnated, with only Czechia and Japan showing declines.

The ROMI for all cancers in 2000–20 across countries are presented in Fig. 2. In these Lexis surfaces, a green diagonal band indicates that a birth cohort experienced lower mortality than the prior cohort at the same ages; a dark diagonal band indicates mortality increases. Figure 2 is largely showing progress in cancer reduction in all eight countries, consistently with the age-standardized rates. However, cancer mortality increased for cohorts born in around 1945 in France, Germany, and Japan. In some countries, increasing cancer mortality also occurred for cohorts born in around 1975, seen most clearly in England & Wales and France. The ROMI in all of these cases appear rather modest, with increases of <5%.

**Figure 2:**
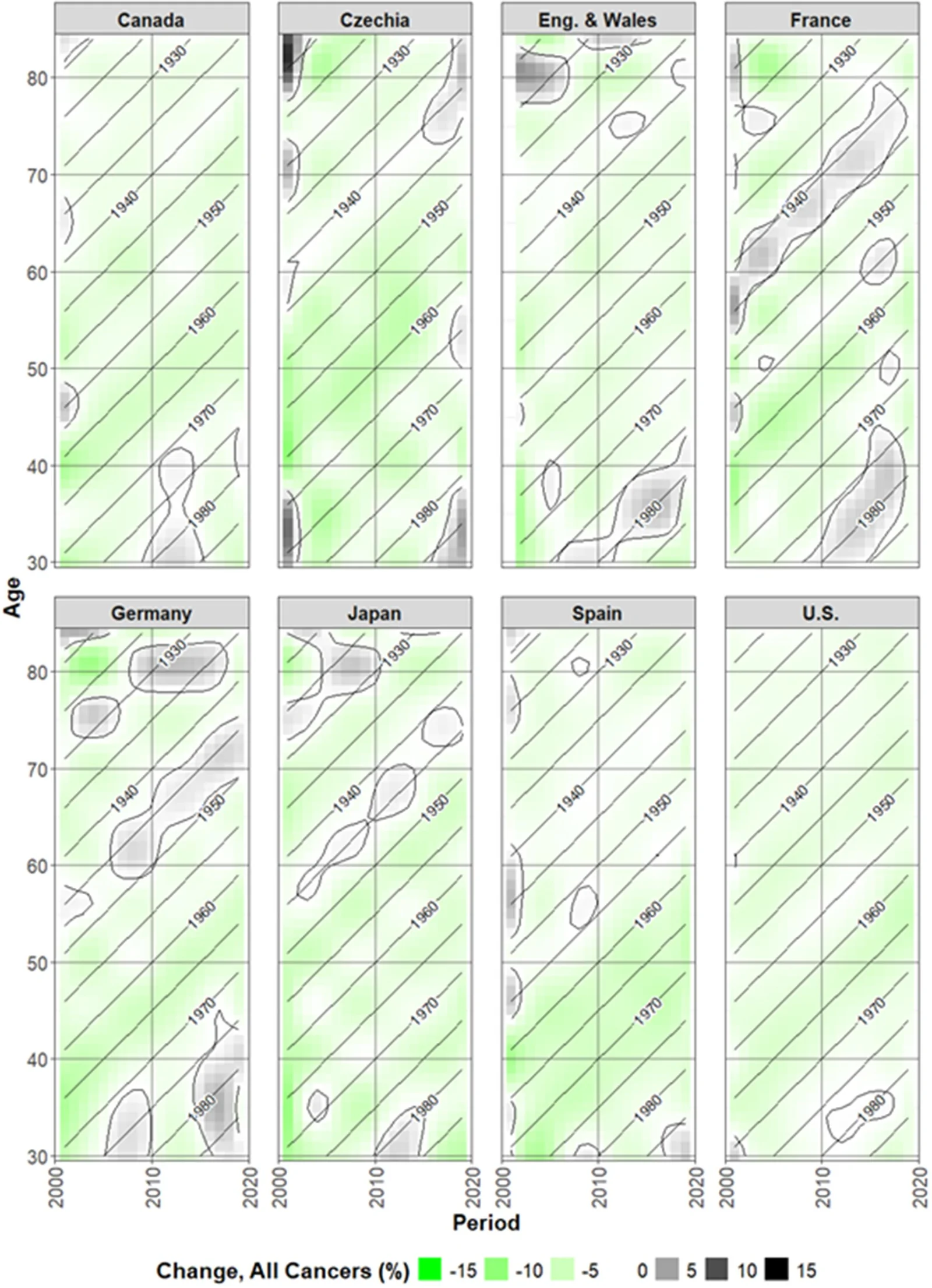
ROMI (%) across eight countries between 2000 and 2020 for all cancers. Data from HCD.

As shown in Fig. 3, lung and larynx cancer has largely been declining across these eight countries, especially for younger birth cohorts. Canada and the USA exhibit notable improvements in lung and larynx cancer across most ages and birth cohorts, with some stagnation in declines for the 1950–60 birth cohorts. There are pronounced increases in mortality for cohorts born in around 1940 in England & Wales, France, Germany, Japan, and Spain. Lung and larynx cancer shows distinct sex-specific patterns: male mortality shows progressive improvements across cohorts in almost all countries, while female mortality shows increases in cohorts born between 1935 and 1955 (Supplementary Figures 4A and 5A).

**Figure 3:**
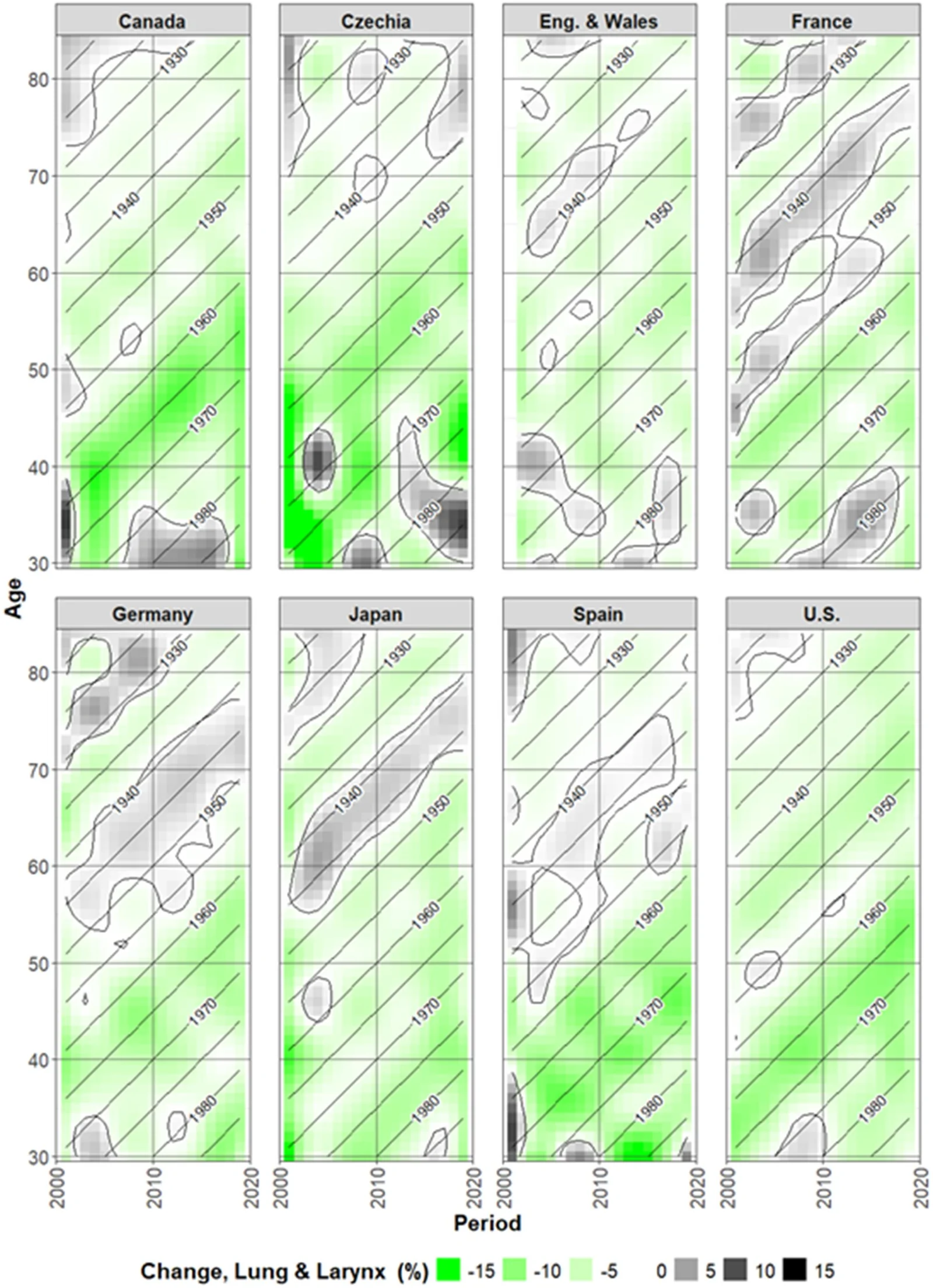
ROMI (%) across eight countries between 2000 and 2020 for lung and larynx cancer. Data from HCD.

Contrary to lung and larynx cancers, improvements in colorectal cancer mortality are concentrated among older birth cohorts (Fig. 4). Canada and the USA exhibit strikingly similar mortality patterns, with decreases in mortality for those born before 1960 and increases for birth cohorts between 1960 and 1985. England & Wales experienced even larger increases for young birth cohorts, at slightly above 10% for people born between 1970 and 1980. Cohorts born after 1970 also experienced increases in colorectal cancer in France and Germany. These results are consistent with those of the two-factor age–cohort sensitivity analyses, with 95% Confidence Intervals (CI) results indicating a robust increase. Period patterns appear in Japan, where colorectal cancer increased across most ages around 2010–15, and in Spain, with increases across ages of ≥50 years in 2005–10. This pattern with earlier and stronger adverse trends in Canada and the USA followed by a similar but temporally lagged trajectory in England & Wales, France, and Germany recurs in both colorectal and liver cancer, suggesting that shared lifestyle transitions spread across continents and cultures with a consistent delay of approximately one decade.

**Figure 4:**
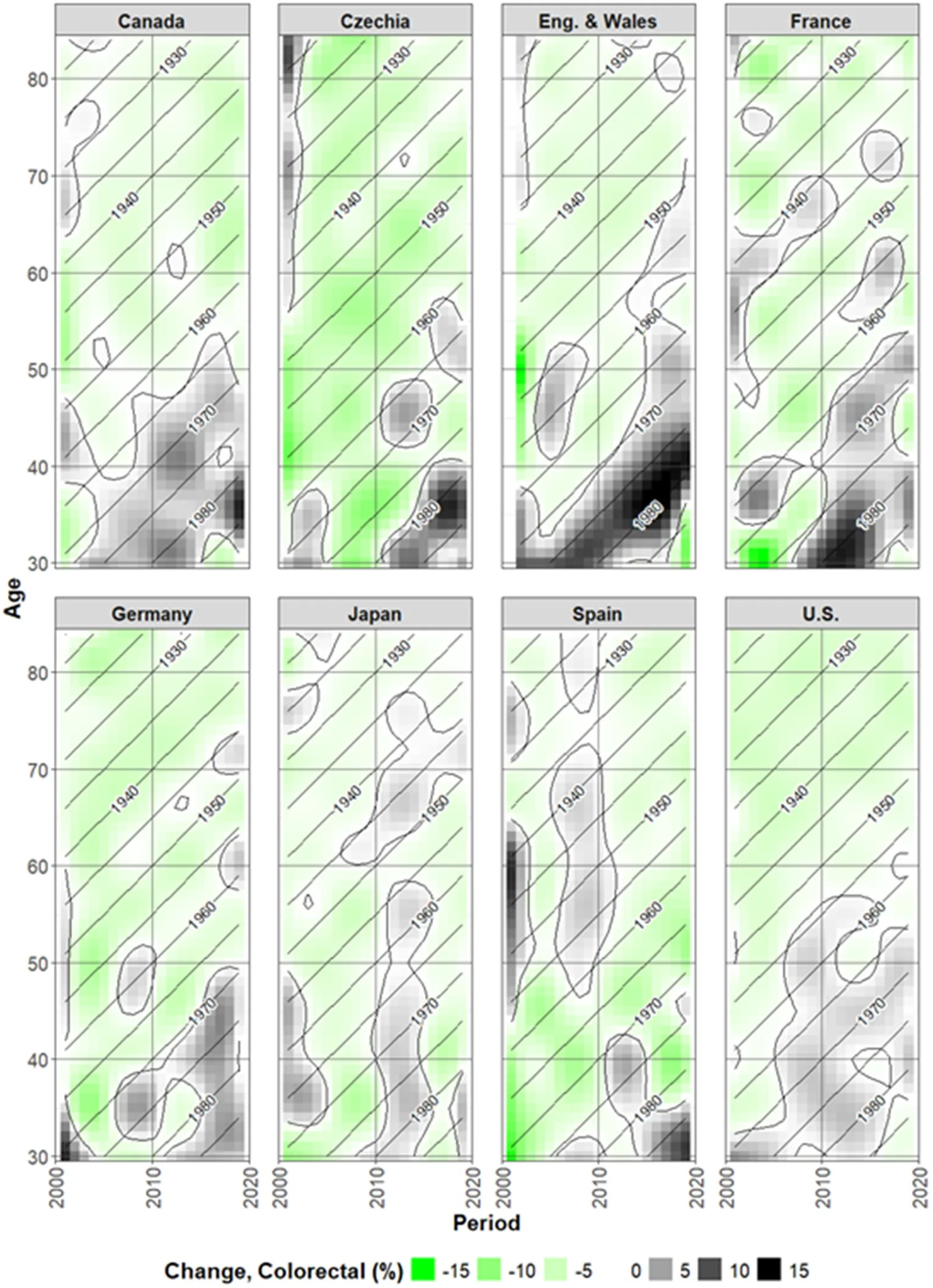
ROMI (%) across eight countries between 2000 and 2020 for colorectal cancer. Data from HCD.

Most countries have experienced declining stomach cancer mortality (Fig. 5). Exceptions include France, which shows increased mortality for the 1970–80 birth cohorts, also seen to a lesser extent in England & Wales, Germany, and the USA.

**Figure 5:**
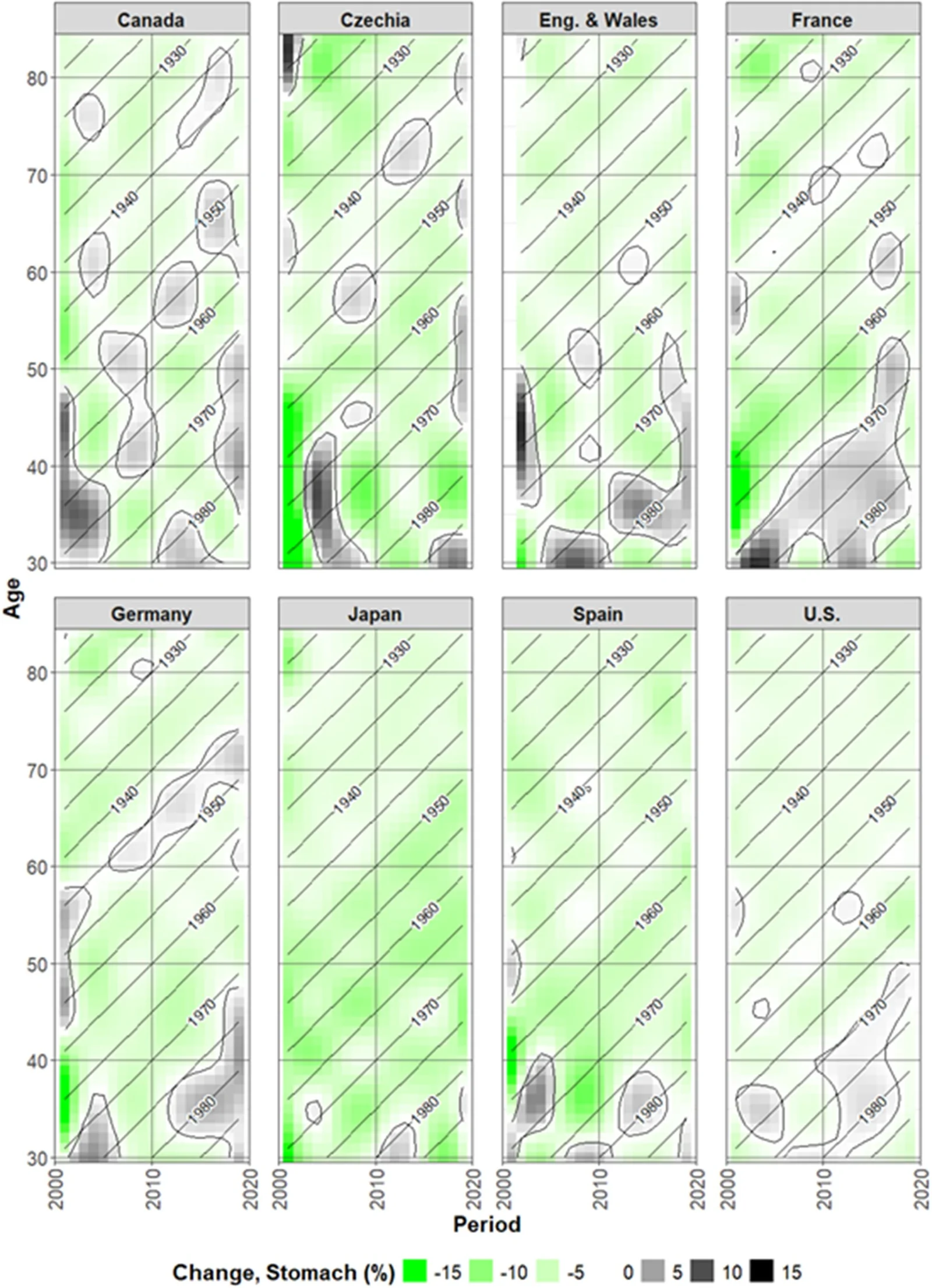
ROMI (%) across eight countries between 2000 and 2020 for stomach cancer. Data from HCD.

Liver cancer mortality (Fig. 6) exhibits strong birth-cohort trends. Canada, England & Wales, Germany, and the USA show worsening liver cancer for older cohorts, with particularly strong increases for cohorts born between 1945 and 1955, nearing 10%, compared with the previous years. In these countries, declines in liver cancer mortality begin around the 1960 birth cohort, but patterns of increasing mortality return among people born in around 1970–80. France shows increasing mortality across several cohorts from the 1920s to the late 1970s, while Spain shows increases for the 1950–60 cohorts. Czechia experienced increases in liver cancer across most ages in 2000–05 and 2015–20, whereas Japan maintained mortality declines across cohorts born after 1930.

**Figure 6:**
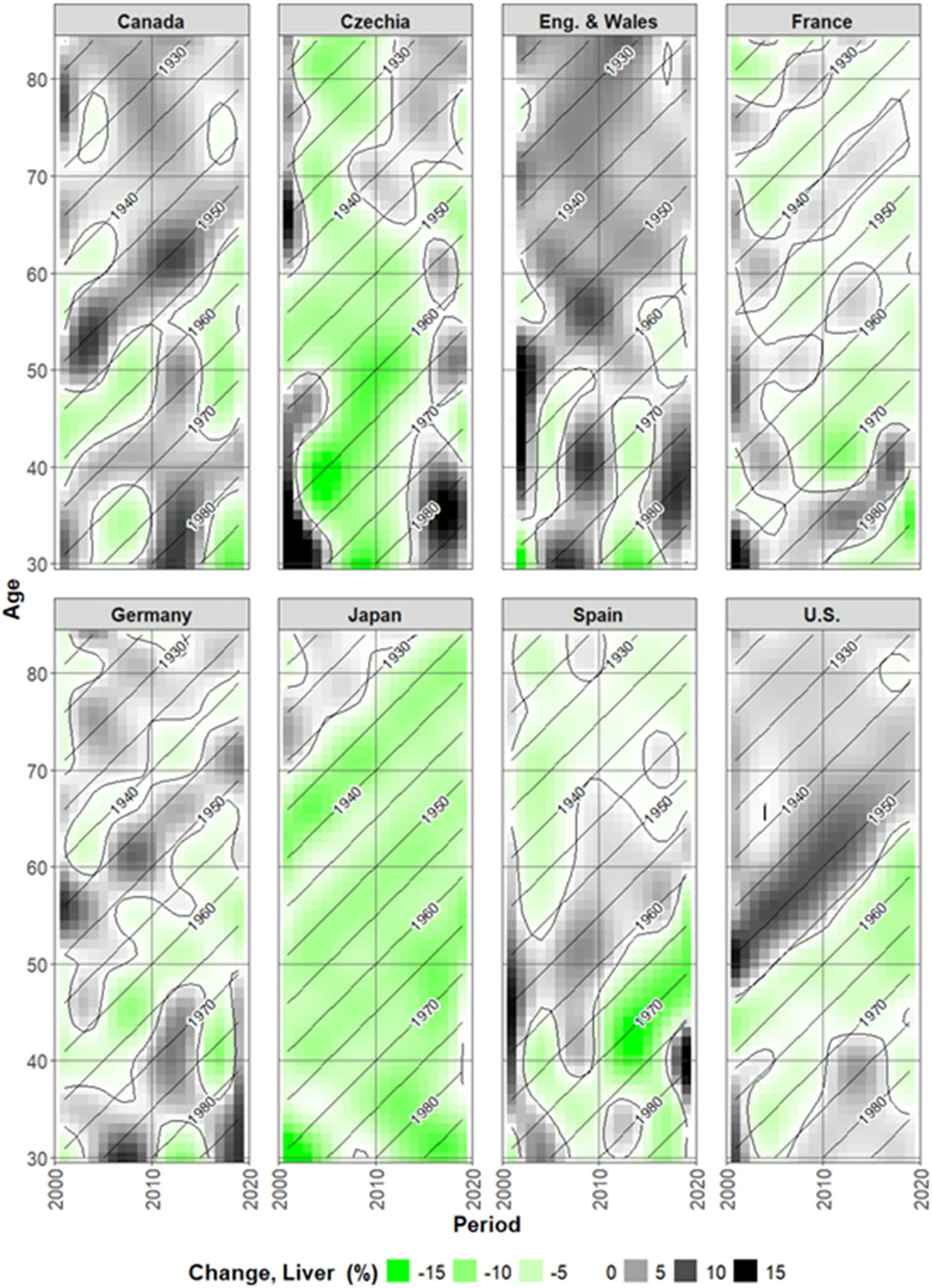
ROMI (%) across eight countries between 2000 and 2020 for liver cancer. Data from HCD.

For other cancers (Supplementary Figure 1A), most countries presented improving trends, with the exception of some birth cohorts in France (1940–45), Germany (1945–50), and Japan (1940–50), which all exhibit slightly increased mortality rates (<4%). Sensitivity analyses using two-factor Poisson models confirmed the magnitude of the observed trends. These models also revealed additional non-linear cohort effects that were not apparent in the Lexis surfaces, particularly in England & Wales for colorectal and liver cancer.

## Discussion

Examining mortality change across birth cohorts reveals distinct generational patterns in mortality from lifestyle-associated cancers across eight high-income countries, with notable variations in magnitude and timing by cancer type and national context. While overall cancer mortality declined in all eight countries, progress is uneven: five of six cancer types showed secular reductions, but liver cancer increased or stagnated in most countries, and worsening colorectal cancer trends in younger cohorts represent an emerging challenge that aggregate statistics obscure.

The patterns observed for lung and larynx cancers align closely with known smoking epidemiology across generations. The pronounced mortality increases for birth cohorts in around 1940–5 in England & Wales, France, Germany, Japan, and Spain reflect the widespread adoption of smoking during and after World War II, when cigarettes were readily available and their health risks were not yet fully understood [5, 7, 9, 10].

More favourable patterns in lung and larynx cancers observed in the USA and Canada, particularly for cohorts born after 1960, likely reflect earlier and more aggressive tobacco-control policies in North America compared with Europe during the latter half of the twentieth century. The stagnation in lung cancer declines for cohorts born between 1950 and 1960 aligns with findings related to higher smoking prevalence for this particular cohort [31].

One key finding from this analysis is the trend of increasing colorectal cancer mortality among people born after 1960 across countries. This finding is consistent with recent reports of increasing colorectal cancer incidence among younger adults in Western countries [14–16]. The strikingly similar patterns in Canada, England & Wales, and the USA support the hypothesis that lifestyle factors (particularly dietary changes, increasing obesity rates, and reduced physical activity) have changed similarly across these populations [12, 13]. In the USA, the per capita high-fructose corn syrup consumption rose by >10-fold from 1970 to 1990 [32]; in Canada, The North American Free Trade Agreement (NAFTA’s) removal of tariffs on the syrup between 1994 and 1998 raised supply and likely consumption [33]. Rapid increases in colorectal cancer mortality in recent birth cohorts in England & Wales suggest that lifestyle-related risk factors are also increasing rapidly among younger adults.

The similar, but delayed, cohort patterns observed in France (1975–80) and Germany (1970–75) indicate that lifestyle transitions associated with colorectal cancer risk may have had a later onset in these European contexts. We do not observe patterns of improvement across all ages over time in a way that suggests a strong role of changing access to screening and treatment, although it is possible that such advances differentially contribute to improvements in specific birth cohorts.

Declining stomach cancer mortality likely reflects improvements in *H. pylori* eradication and food safety [34]. Modest adverse trends in younger cohorts in France and the USA suggest that diet quality and alcohol consumption may partially offset infectious-disease gains. Japan’s convergence toward other countries in stomach and liver cancer rates by 2020 (after being an outlier in 2000) illustrates how aggressively targeted public health measures can reshape cohort trajectories.

The diverse patterns observed for liver cancer reflect the multifactorial nature of this disease, with contributions from viral hepatitis, alcohol consumption, obesity, and other lifestyle factors [18, 35]. The particularly high ROMI for cohorts born between 1945 and 1960 in Canada, England & Wales, Germany, and the USA likely reflect a combination of factors, including exposure to hepatitis viruses during periods of limited screening, as well as increased alcohol and tobacco consumption among these generations [36, 37]. The worsening patterns for more recent cohorts align with recent reports of increasing liver cancer mortality between 2010 and 2019 [19] and may reflect the rising prevalence of non-alcoholic fatty liver disease associated with obesity and metabolic syndrome.

Period patterns observed in some countries (most notably the colorectal cancer increase across most ages in Japan in around 2010–15 and in Spain in around 2005–10) warrant cautious interpretation. Vertical bands in these Lexis surfaces affecting all age groups simultaneously are more consistent with changes in cancer-registration practices or shifts in underlying-cause attribution than with genuine epidemiological change, as such artefacts would be expected to affect all ages uniformly within a given year rather than following the diagonal cohort trajectories that characterize the findings reported here [26, 28].

A key contribution of this study is that aggregate age-standardized rates (as shown in Fig. 1) can mask adverse patterns that only become visible when mortality is examined through a cohort lens. Overall cancer mortality declined in all eight countries—an encouraging story, yet cohort patterns reveal a more concerning picture among younger birth cohorts [23]. Lexis surfaces sidestep this problem by allowing visualization without imposing a functional form. This approach has been applied to all-cause and cause-specific mortality in single-country settings [24, 25], but had not previously been extended to a systematic cross-national comparison of lifestyle-associated cancers. The two-factor Poisson sensitivity analyses corroborate the cohort patterns without incurring the identification costs of full statistical modelling. Combining visual and statistical approaches provides a more comprehensive description of cancer mortality trends.

### Limitations

This study has noteworthy limitations. Plotting mortality data does not capture improvements in cancer survival that may be simultaneous with changing incidence patterns. The 20-year observation period may not have fully captured the long-term patterns for cancers with long latency. Death counts are provided in 4-year groups, requiring interpolation to single ages; sensitivity analyses with unsmoothed data confirmed that our findings are robust to this choice. Future analyses incorporating incidence data and linking patterns to specific policy interventions would enrich interpretation.

The close alignment of Canada, England & Wales, and the USA; the partial alignment of France, Germany, and Spain; and the consistent distinctiveness of Japan point to shared lifestyle environments and policy contexts as primary drivers of generational cancer risk. The worsening colorectal and liver cancer burdens in younger birth cohorts indicate that lifestyle-related cancer risks are increasing among adults born after 1970 in several high-income countries, despite aggregate improvements in overall cancer mortality. Cancer-prevention and early-detection strategies need to be adapted to these generational risk profiles as these cohorts experience increasing cancer risk with age in the coming decades.

## Ethics approval

Not required; this study used de-identified, publicly available data [45 CFR §46.101(b)(4)].

## Supplementary Material

dyag198_Supplementary_Data

## Data Availability

The data supporting this study are publicly available from the Human Cause of Death Database (https://www.mortality.org/Data/HCD). Analytic code is available at https://osf.io/dtvbf.
