# Supplementary material for: Evolution of birth-cohort patterns in lifestyle-associated cancer mortality across high-income countries
Source: Int J Epidemiol. 2026 Oct 6;55(5):dyag198. doi: 10.1093/ije/dyag198 (PMC13641068; doi:10.1093/ije/dyag198)
Supplement: Supplementary file 1 [file dyag198_supplementary_data.zip › ije-2026-05-0861-File009.pdf]

Supplementary material

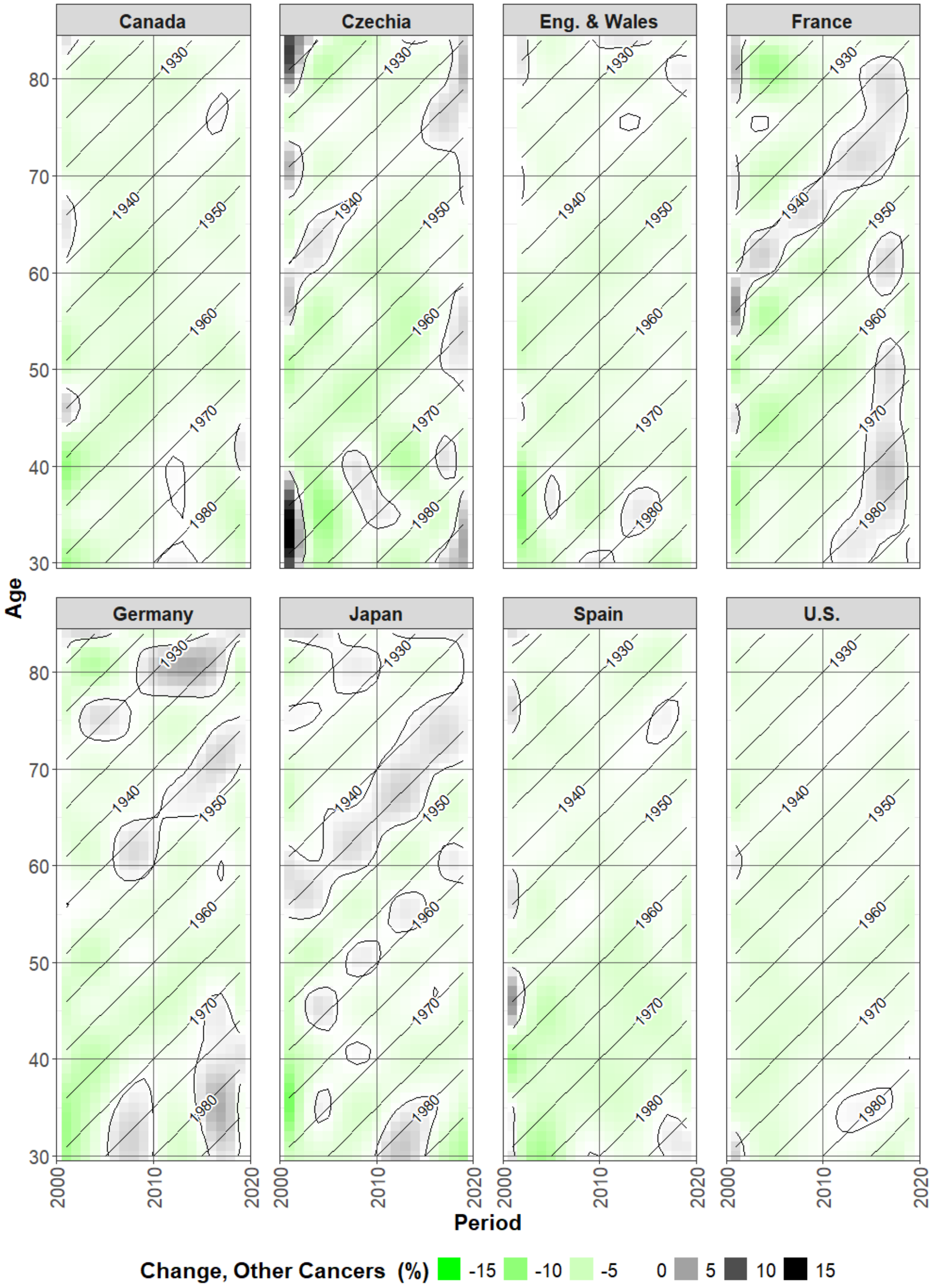

Figure A1: Rates of Mortality Improvement (%) across eight countries between 2000–2020, All Other Cancers. Data from Human Cause of Death Data Series

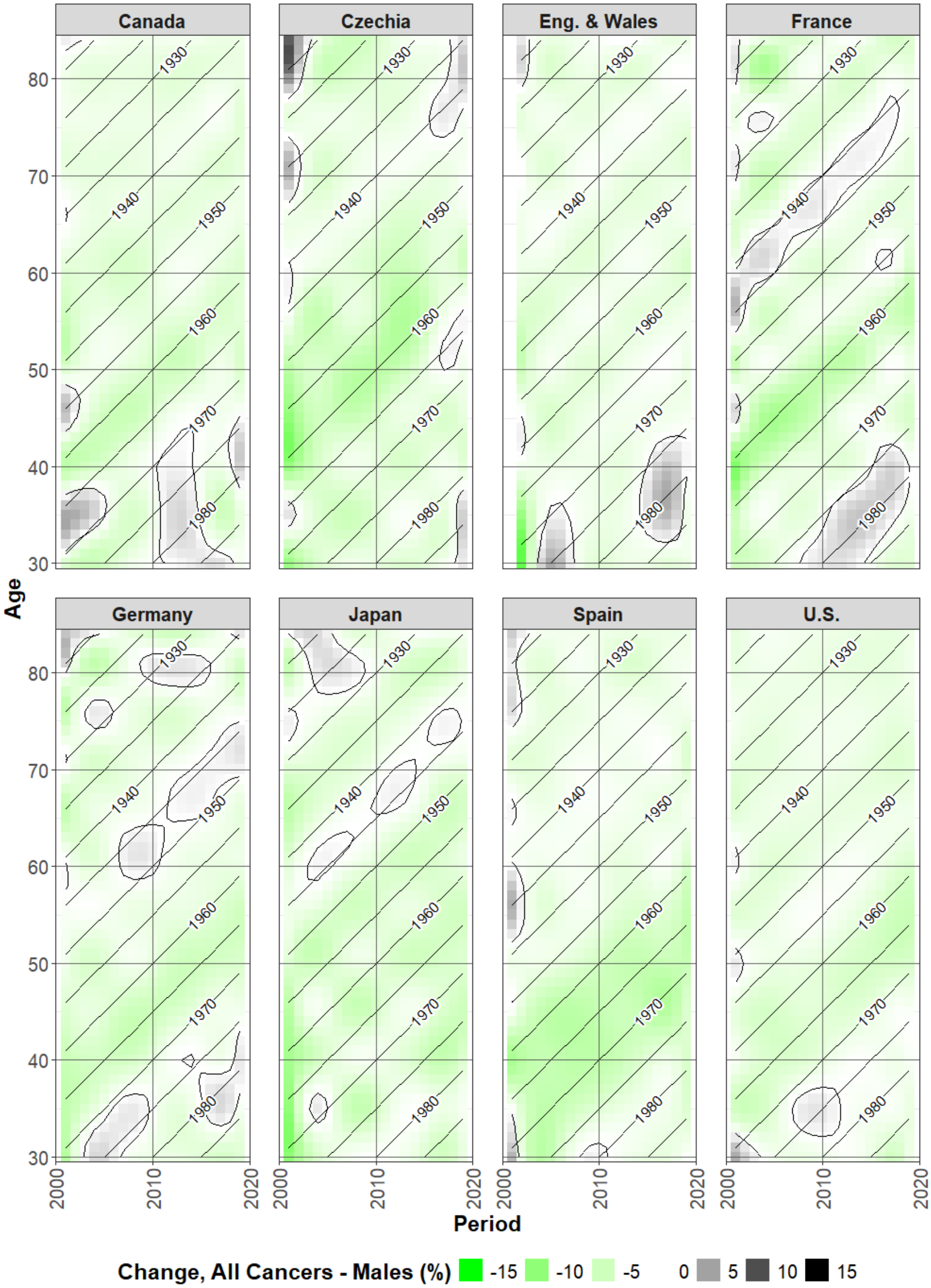

Figure A2: Rates of Mortality Improvement (%) across eight countries between 2000-2020, All Cancers, Males. Data from Human Cause of Death Data Series.

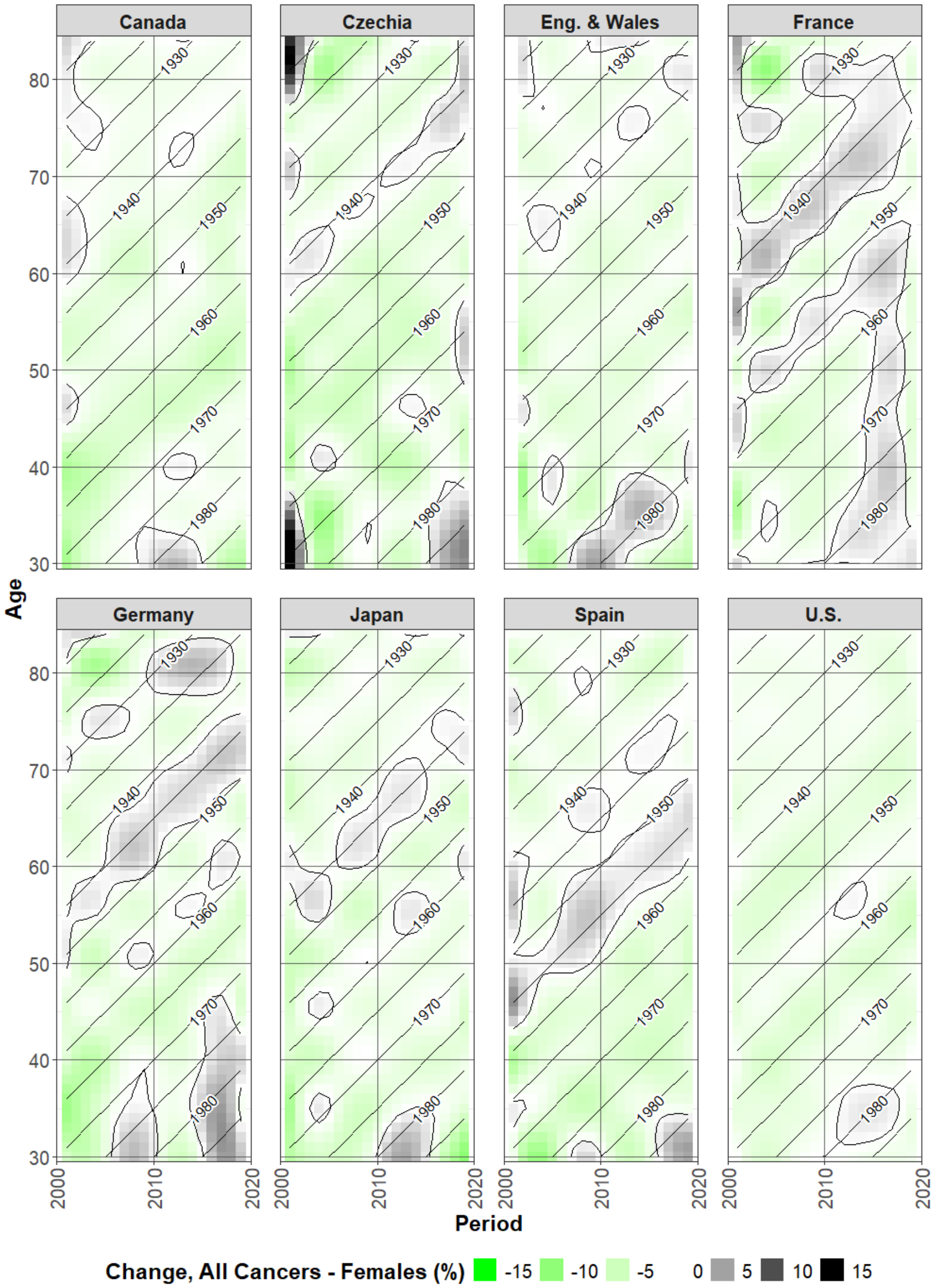

Figure A3: Rates of Mortality Improvement (%) across eight countries between 2000-2020, All Cancers, Females. Data from Human Cause of Death Data Series.

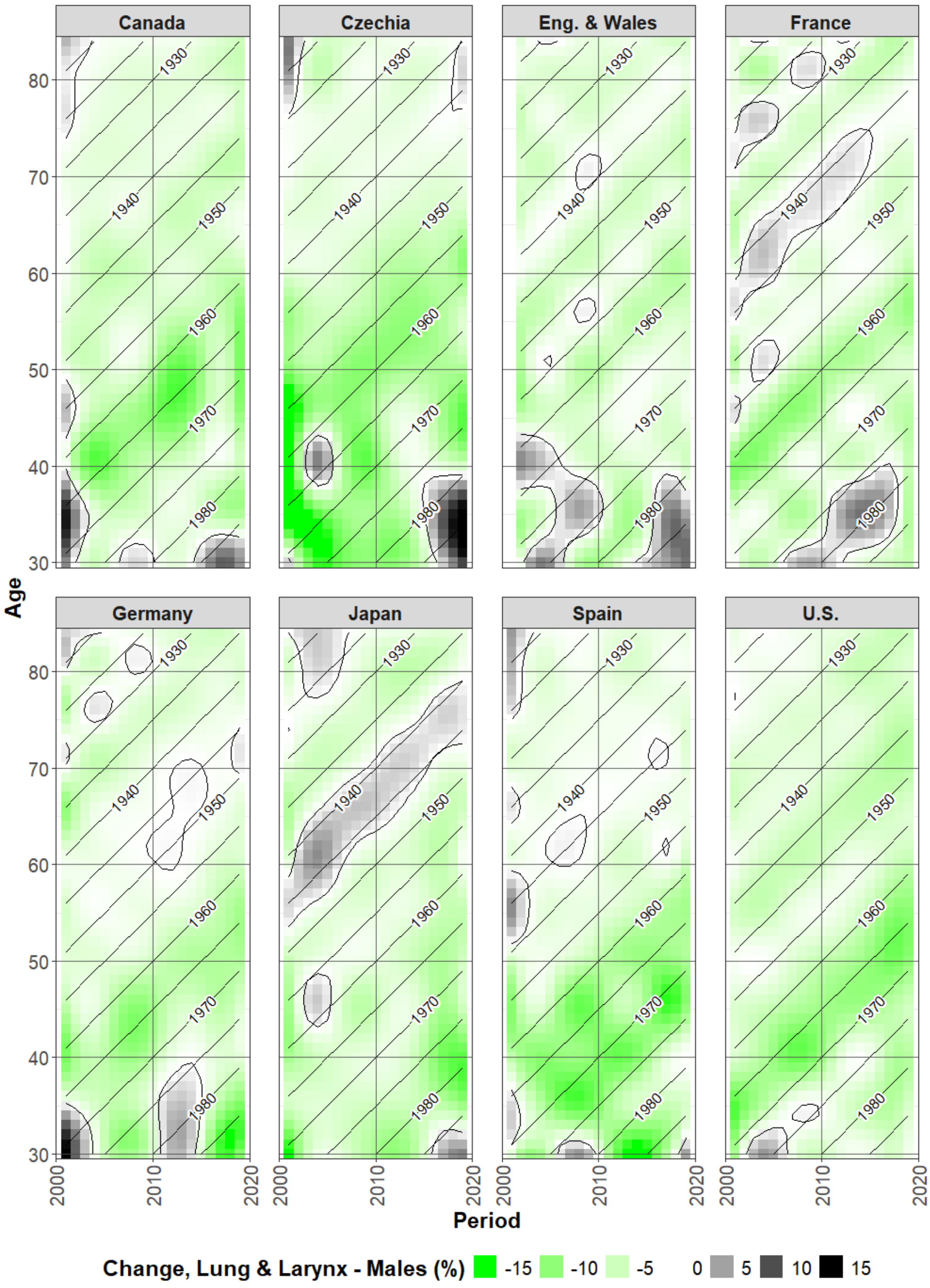

Figure A4: Rates of Mortality Improvement (%) across eight countries between 2000-2020, Lung & Larynx Cancer, Males. Data from Human Cause of Death Data Series.

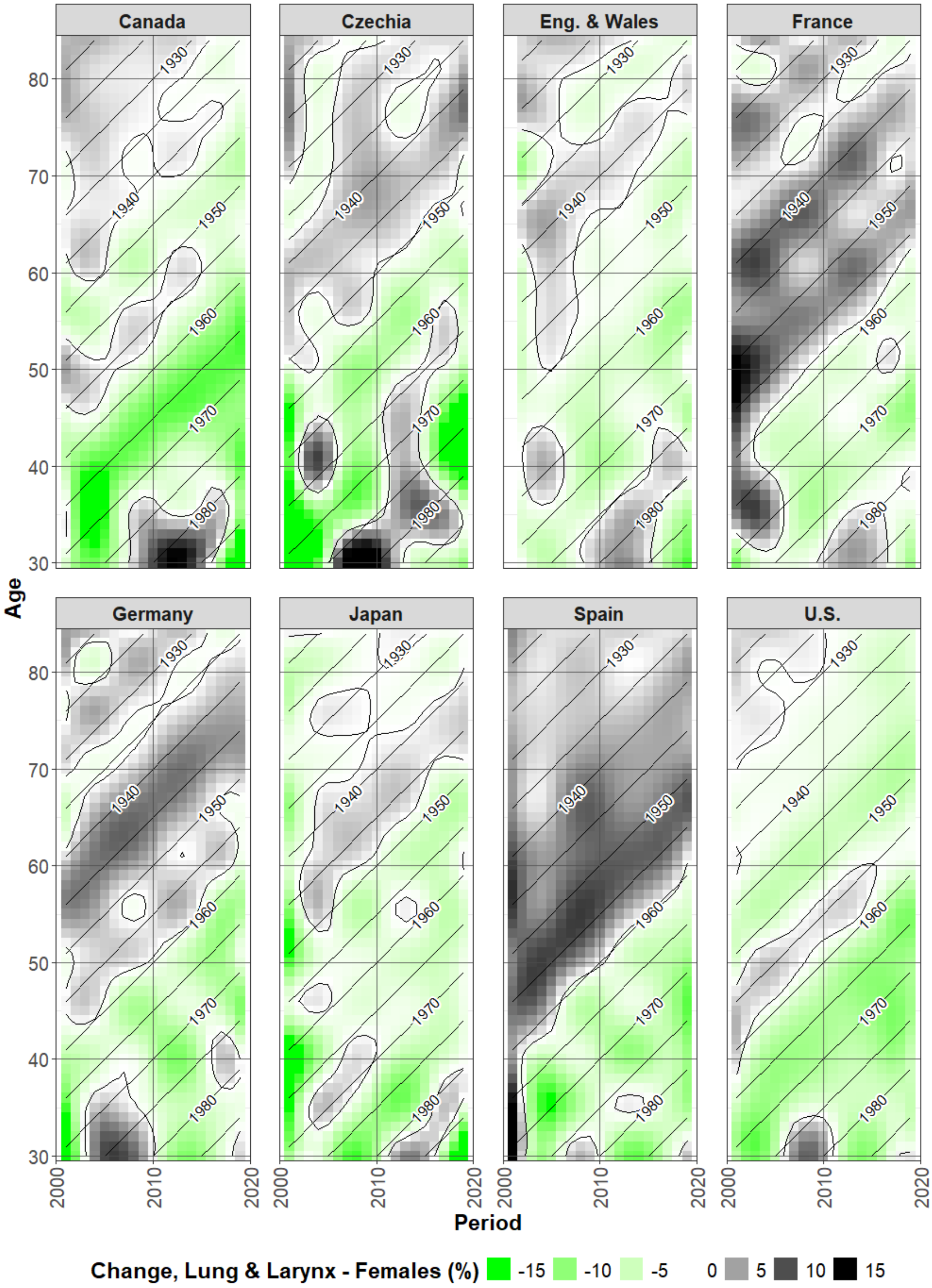

Figure A5: Rates of Mortality Improvement (%) across eight countries between 2000-2020, Lung & Larynx Cancer, Females. Data from Human Cause of Death Data Series.

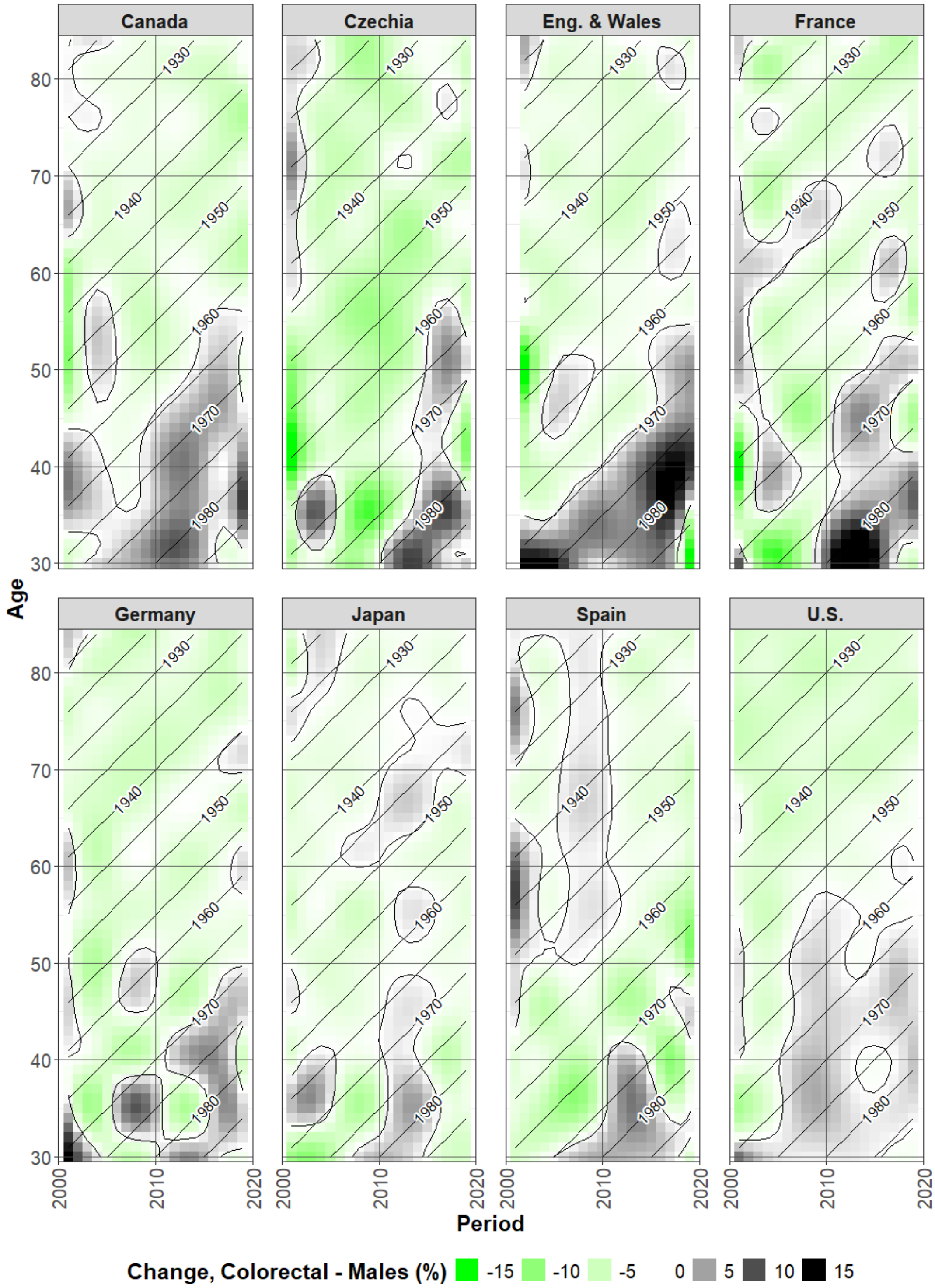

Figure A6: Rates of Mortality Improvement (%) across eight countries between 2000-2020, Colorectal Cancer, Males. Data from Human Cause of Death Data Series.

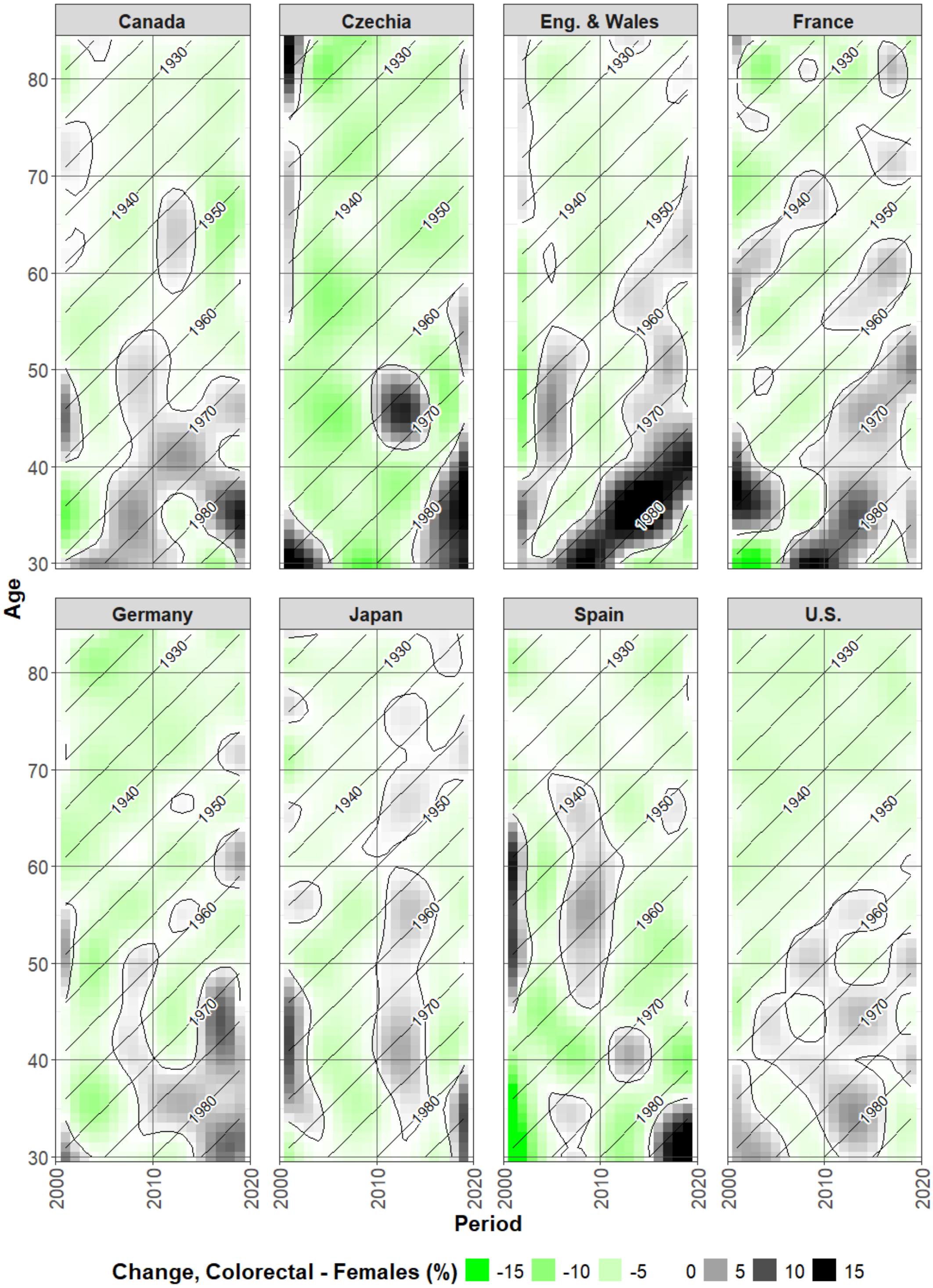

Figure A7: Rates of Mortality Improvement (%) across eight countries between 2000-2020, Colorectal Cancer, Females. Data from Human Cause of Death Data Series.

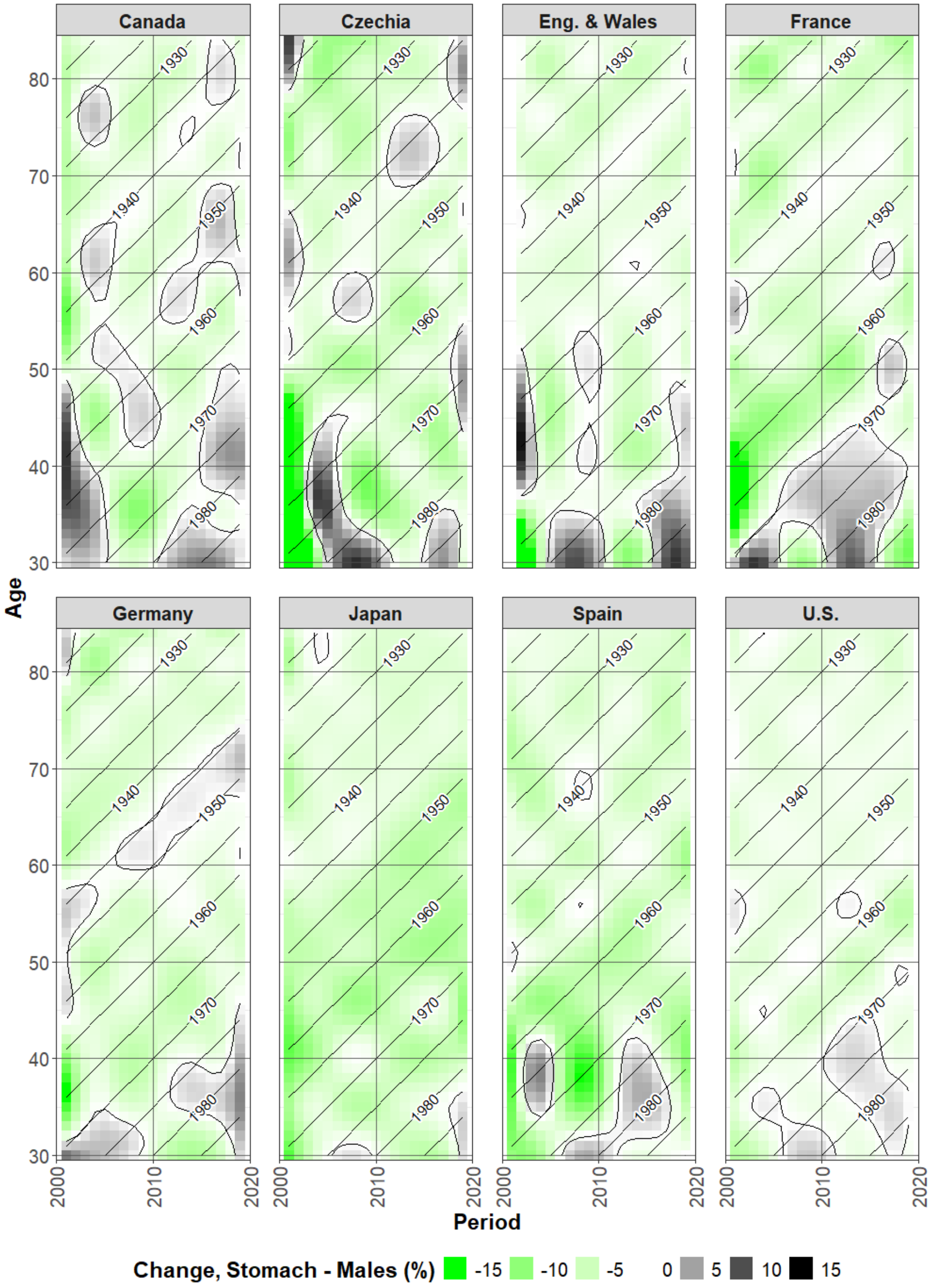

Figure A8: Rates of Mortality Improvement (%) across eight countries between 2000-2020, Stomach Cancer, Males. Data from Human Cause of Death Data Series.

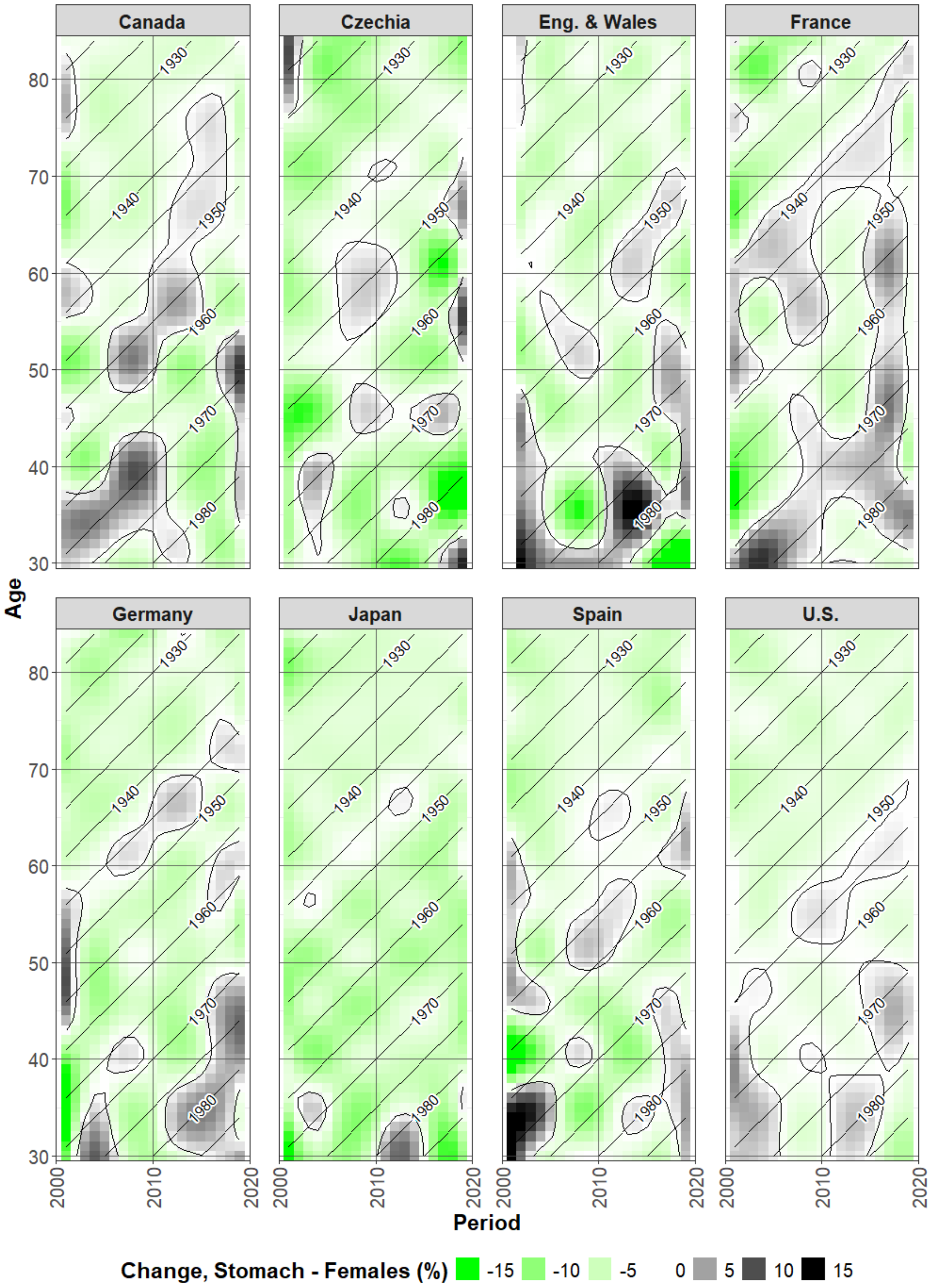

Figure A9: Rates of Mortality Improvement (%) across eight countries between 2000-2020, Stomach Cancer, Females. Data from Human Cause of Death Data Series.

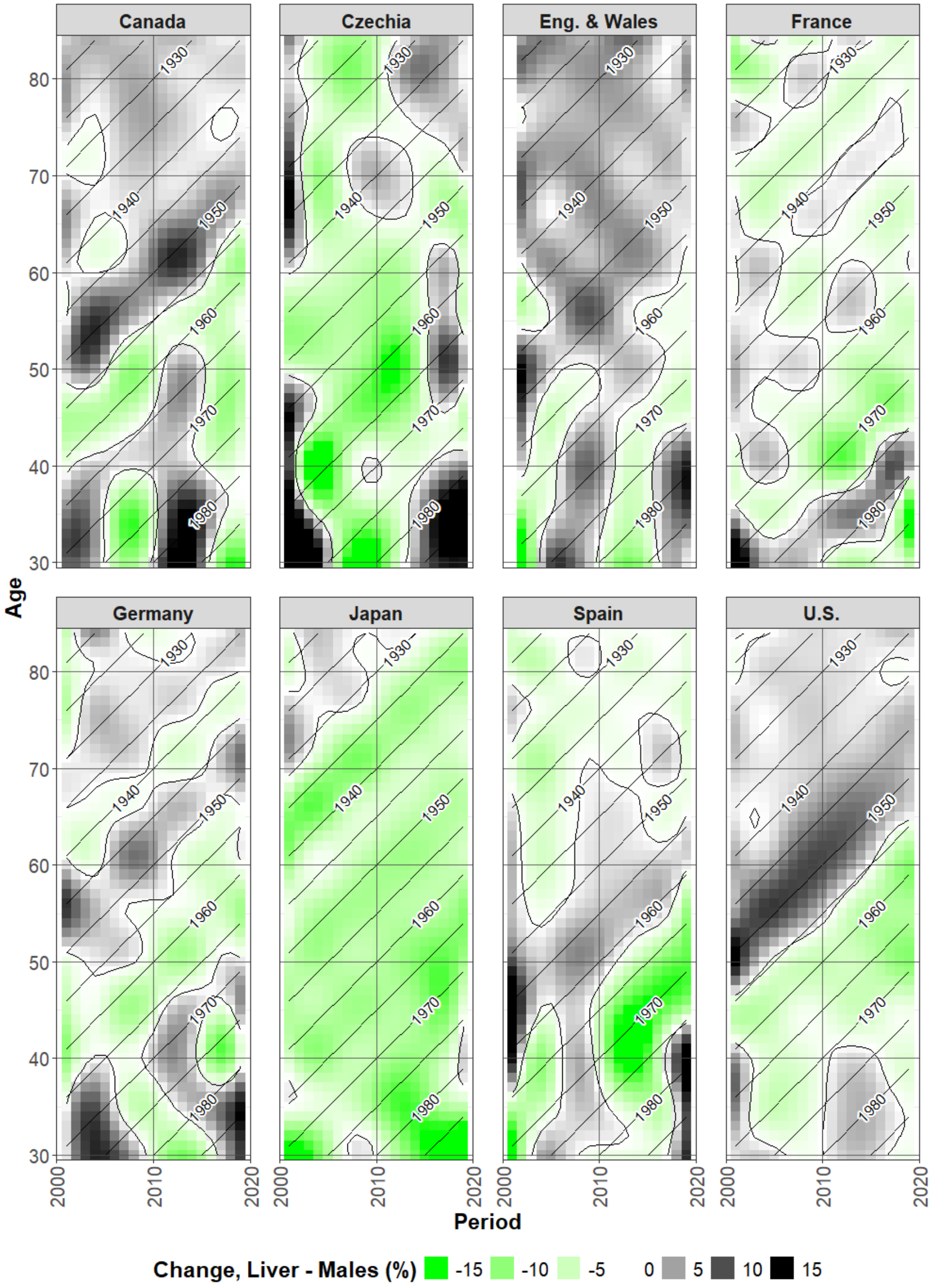

Figure A10: Rates of Mortality Improvement (%) across eight countries between 2000-2020, Liver Cancer, Males. Data from Human Cause of Death Data Series.

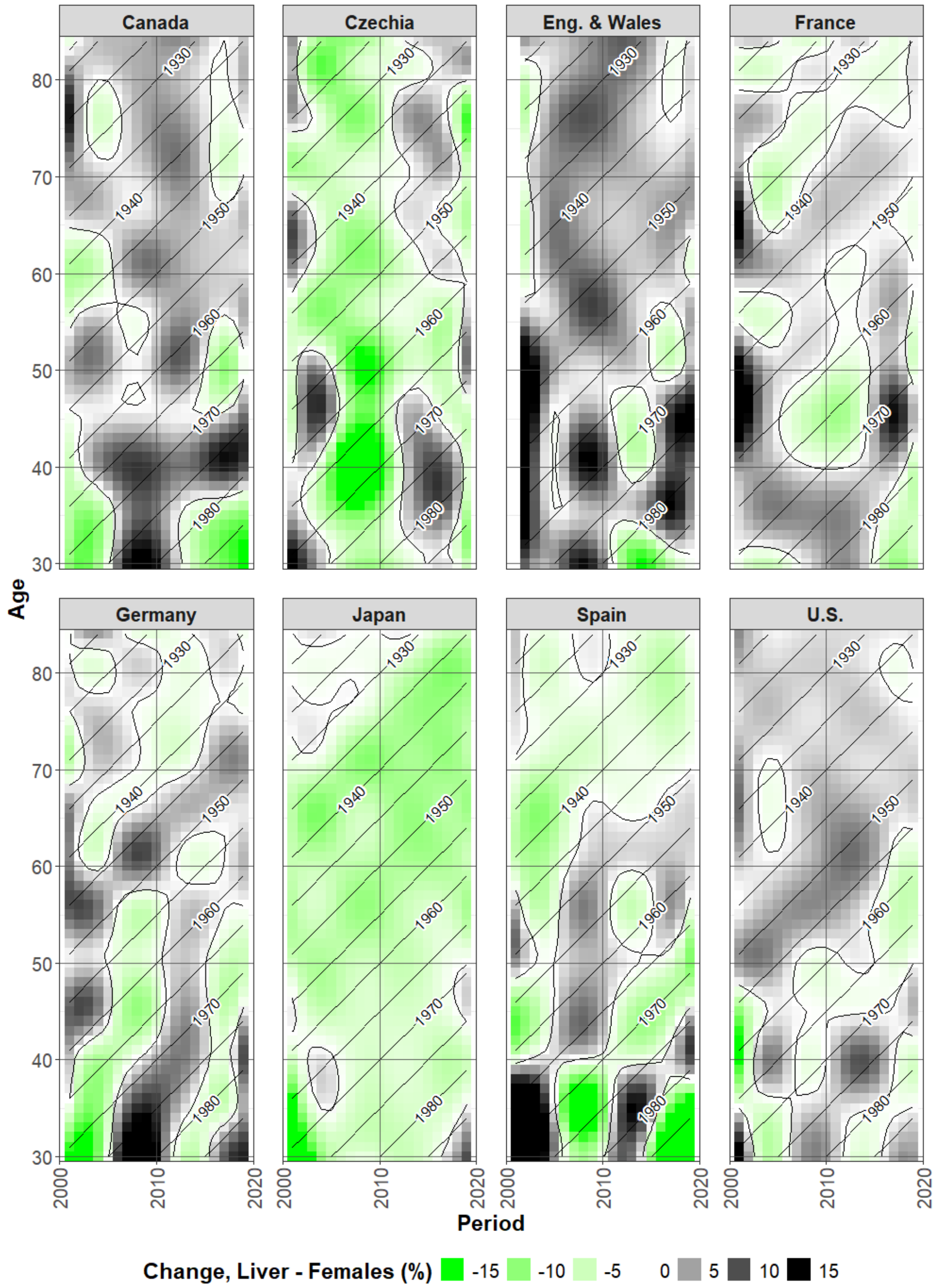

Figure A11: Rates of Mortality Improvement (%) across eight countries between 2000-2020, Liver Cancer, Females. Data from Human Cause of Death Data Series.

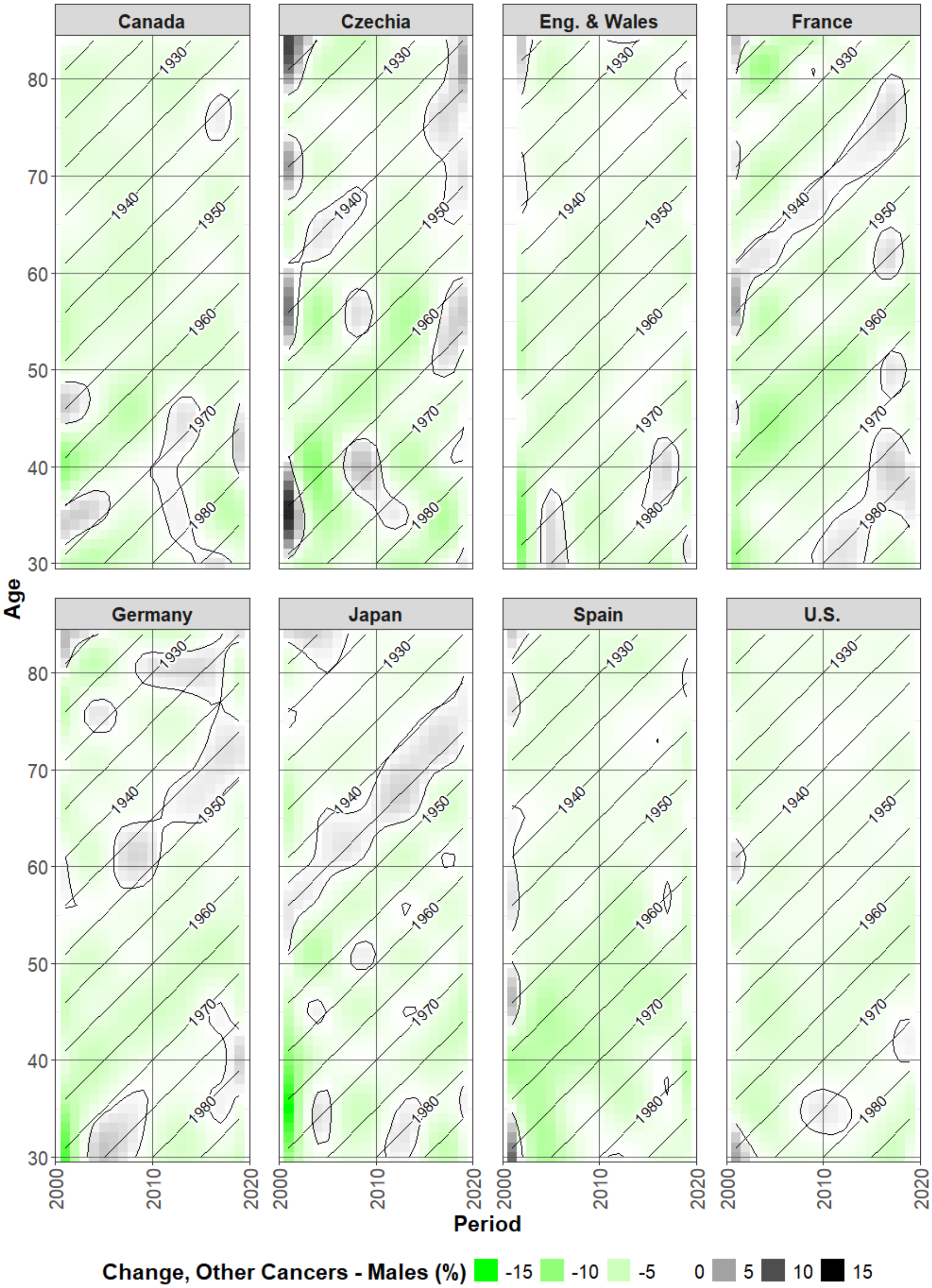

Figure A12: Rates of Mortality Improvement (%) across eight countries between 2000-2020, All Other Cancers, Males. Data from Human Cause of Death Data Series.

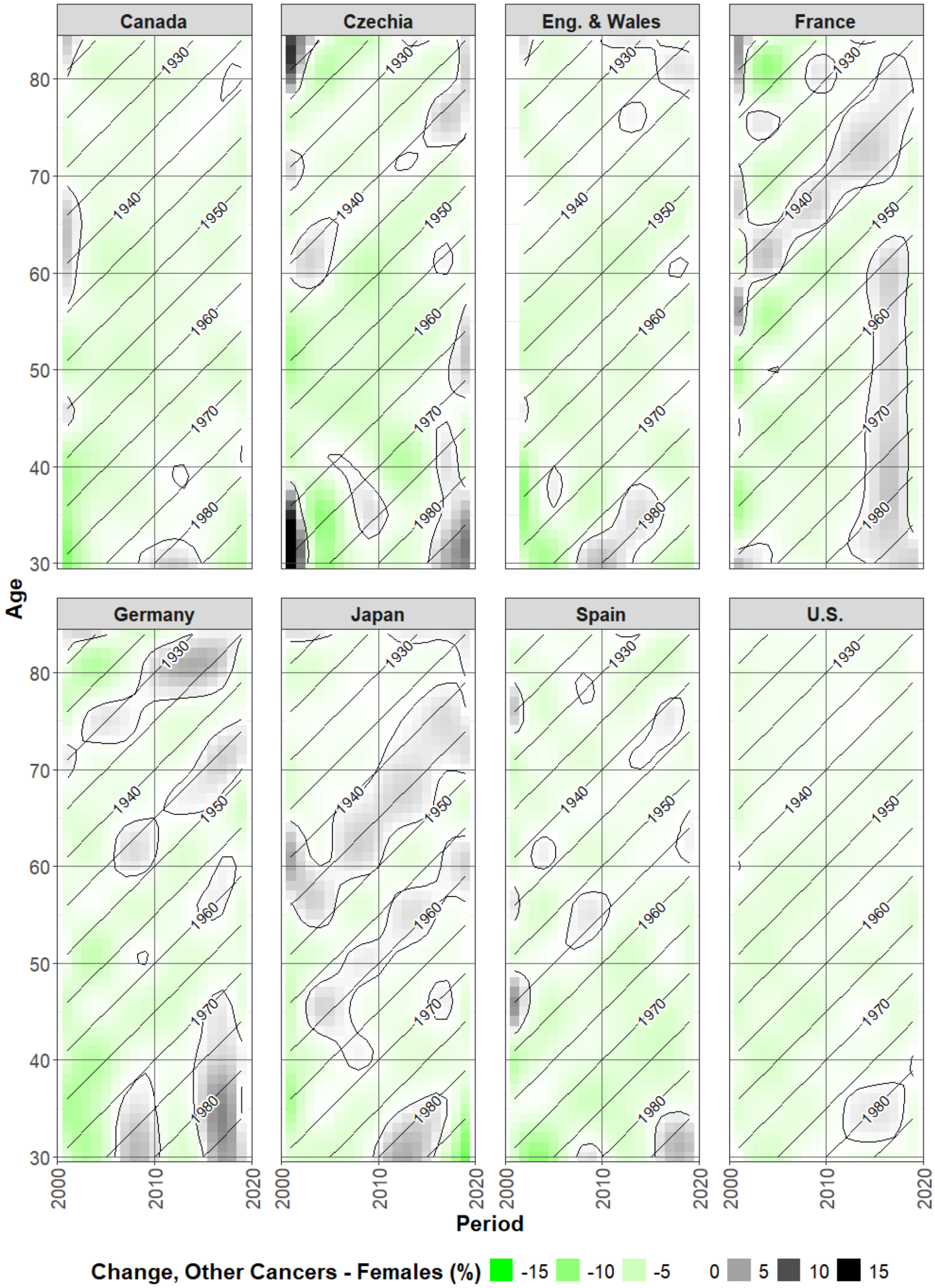

Figure A13: Rates of Mortality Improvement (%) across eight countries between 2000-2020, All Other Cancers, Females. Data from Human Cause of Death Data Series.

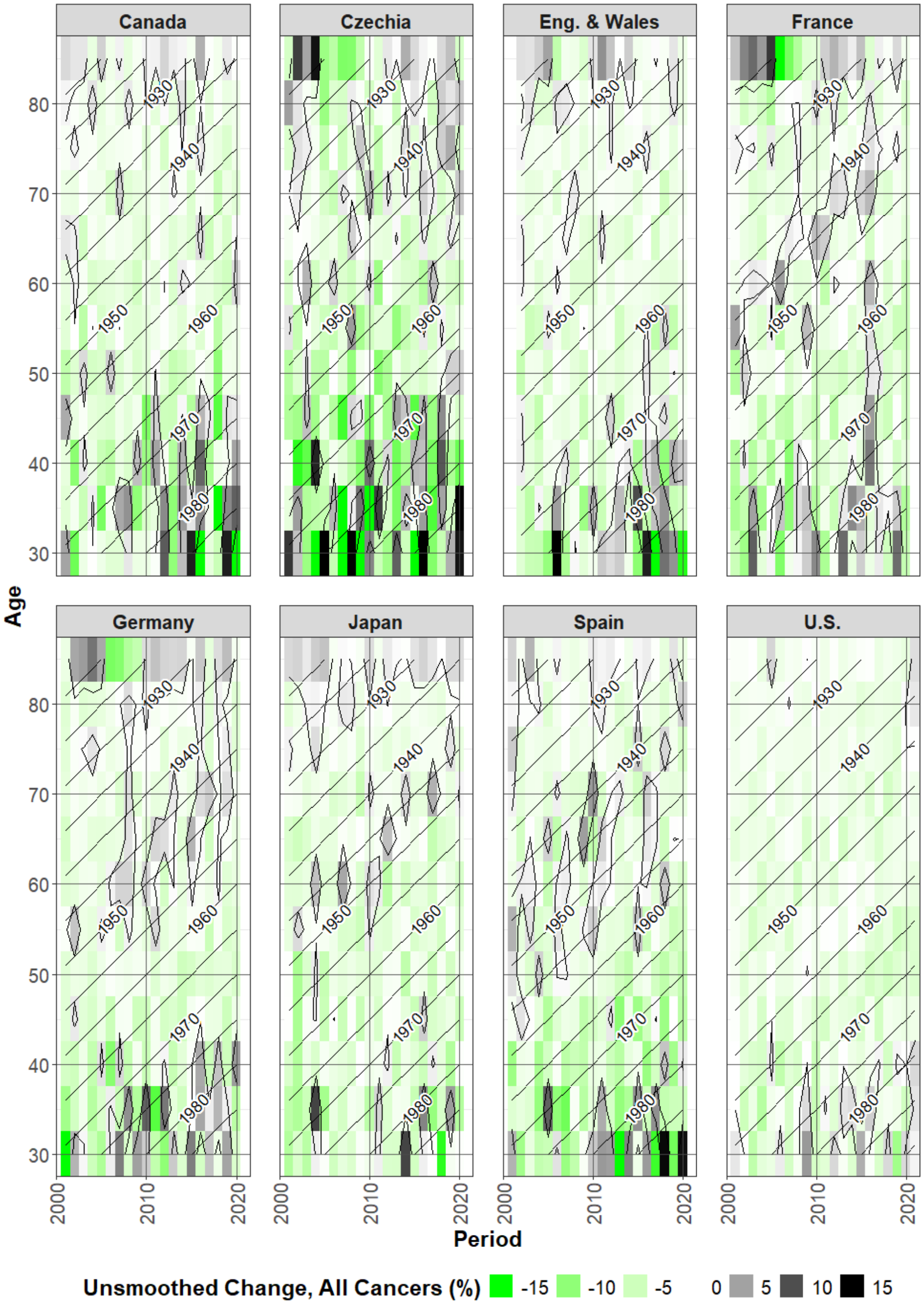

Figure A14: Unsmoothed Rates of Mortality Improvement (%) across eight countries between 2000-2020, All Cancers. Data from Human Cause of Death Data Series.

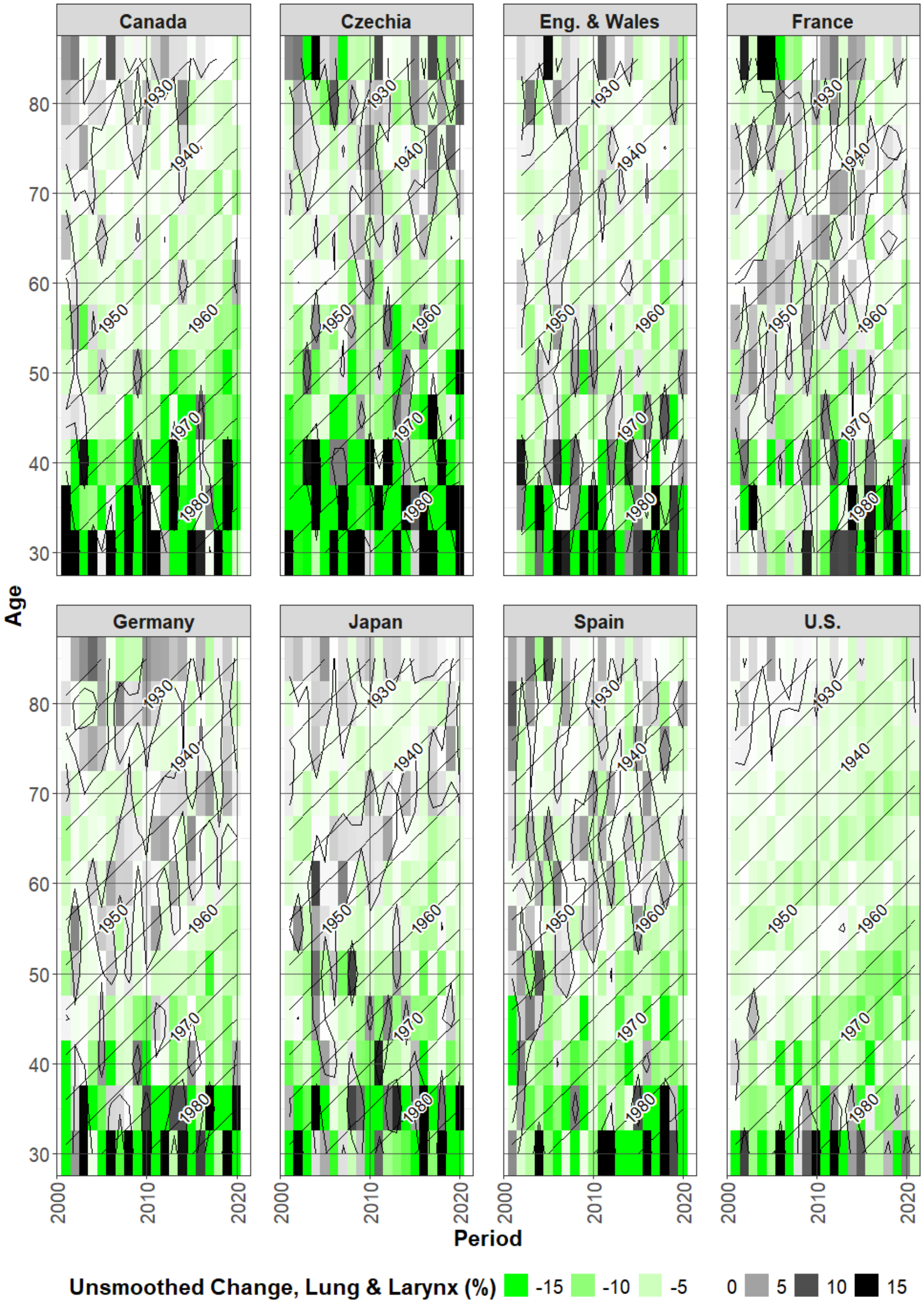

Figure A15: Unsmoothed Rates of Mortality Improvement (%) across eight countries between 2000-2020, Lung & Larynx Cancer. Data from Human Cause of Death Data Series.

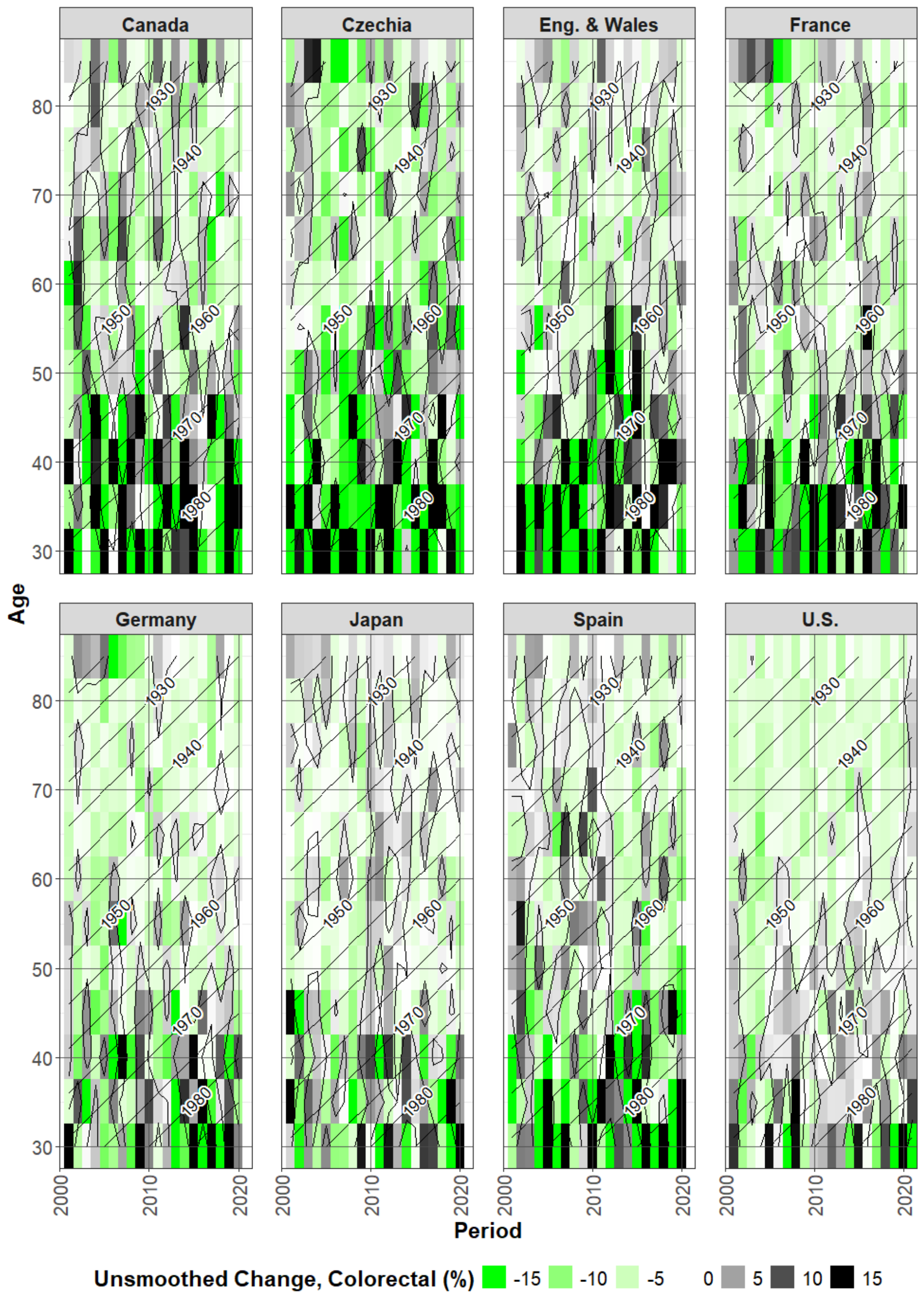

Figure A16: Unsmoothed Rates of Mortality Improvement (%) across eight countries between 2000-2020, Colorectal Cancer. Data from Human Cause of Death Data Series.

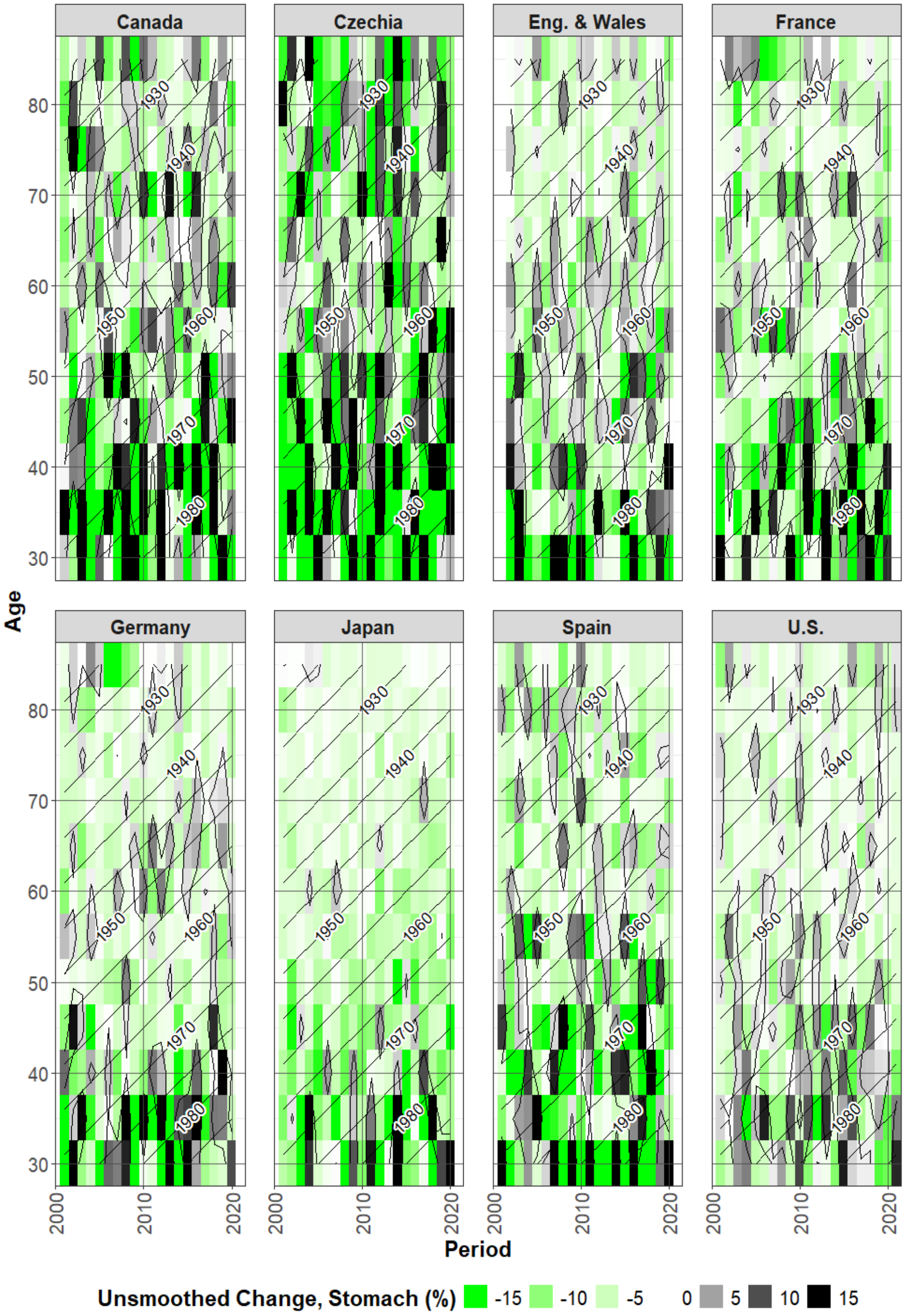

Figure A17: Unsmoothed Rates of Mortality Improvement (%) across eight countries between 2000-2020, Stomach Cancer. Data from Human Cause of Death Data Series.

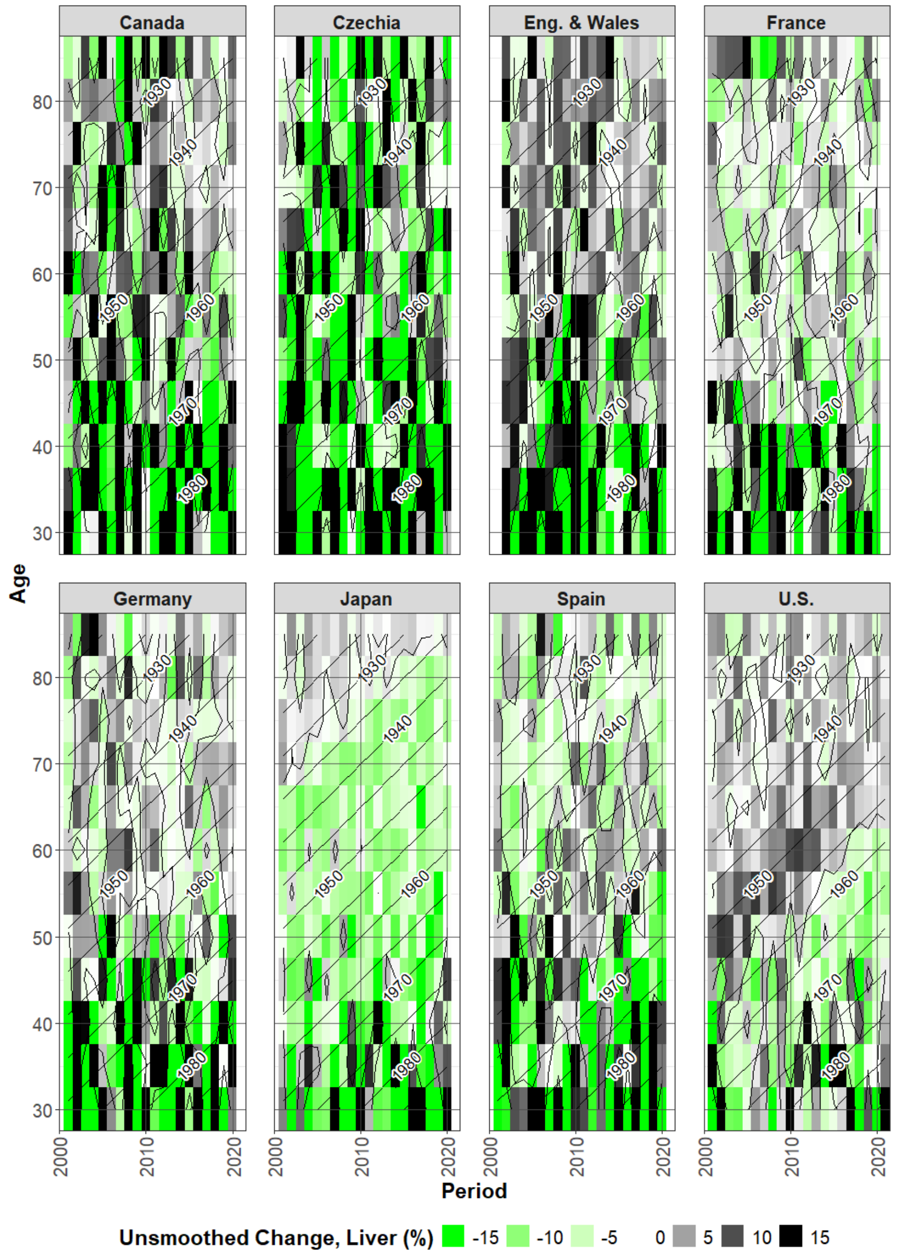

Figure A18: Unsmoothed Rates of Mortality Improvement (%) across eight countries between 2000-2020, Liver Cancer. Data from Human Cause of Death Data Series.

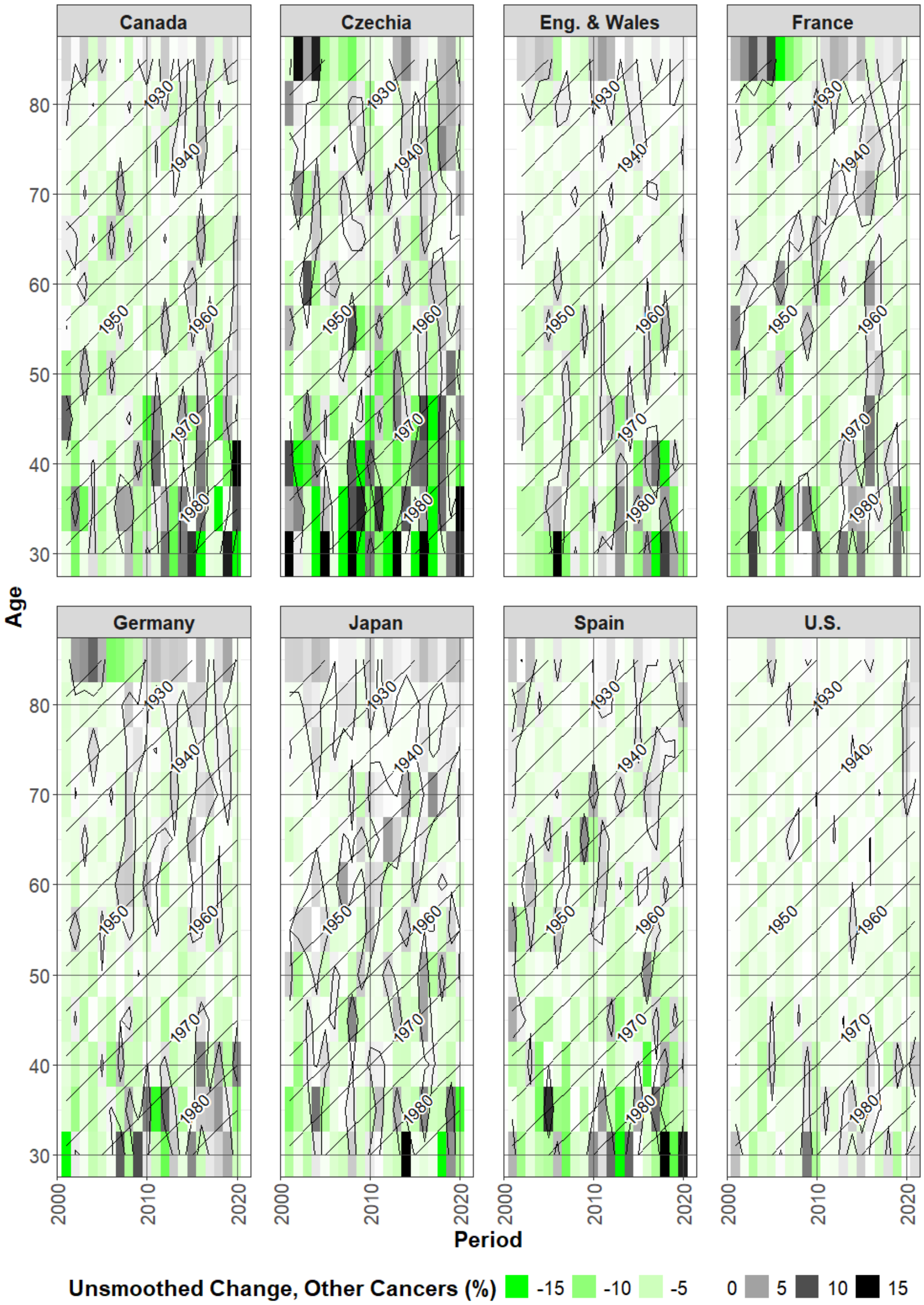

Figure A19: Unsmoothed Rates of Mortality Improvement (%) across eight countries between 2000-2020, Other Cancers. Data from Human Cause of Death Data Series.

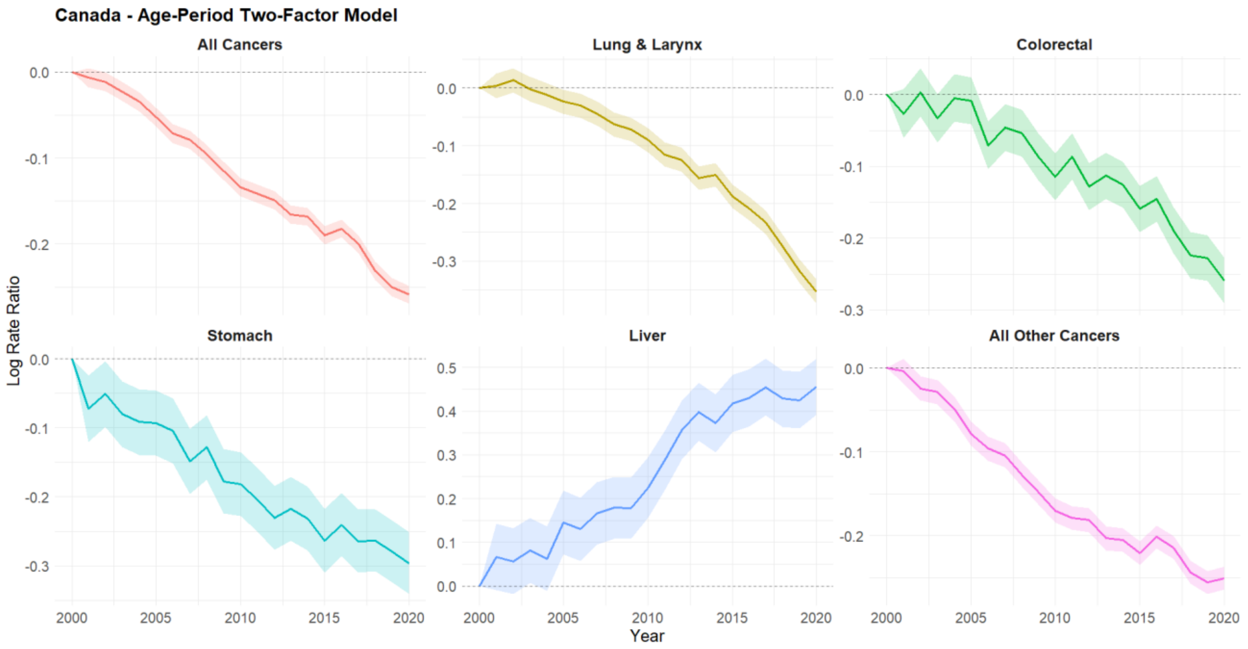

Figure A20: Log-linear cancer disease mortality rates for two-factor Age–Period models by year and type, Canada, 2000–2020.

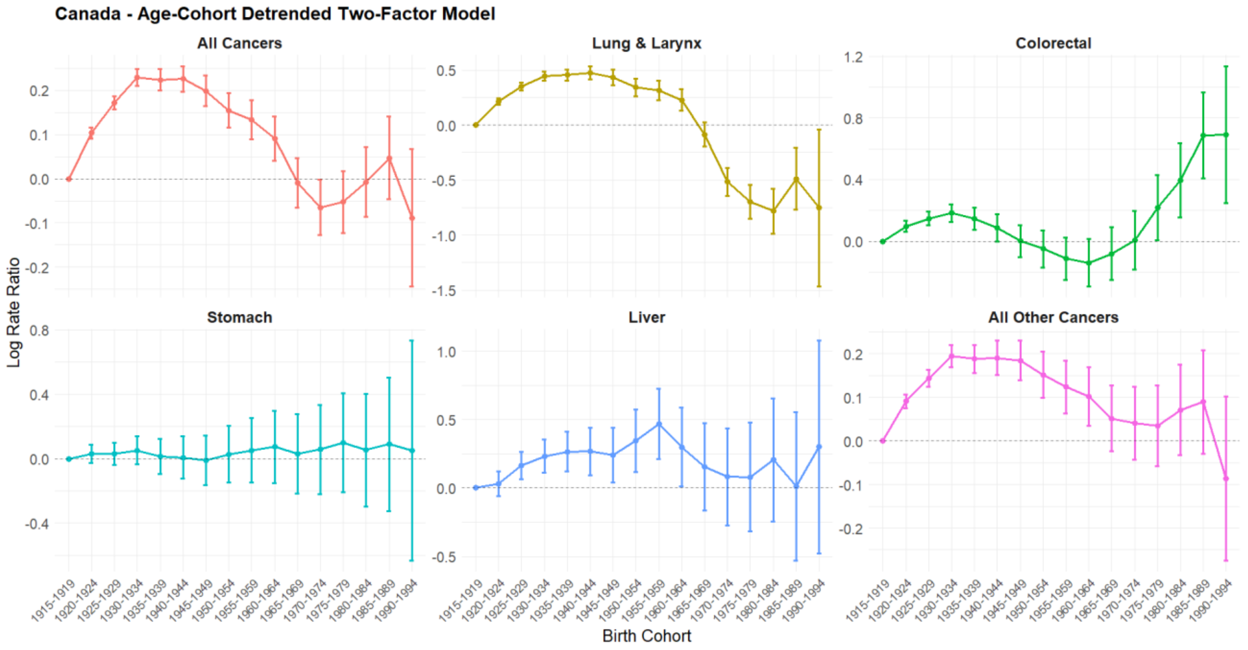

Figure A21: Log-linear cancer disease mortality rates for two-factor Age–Cohort models by year and type, Canada, 2000–2020.

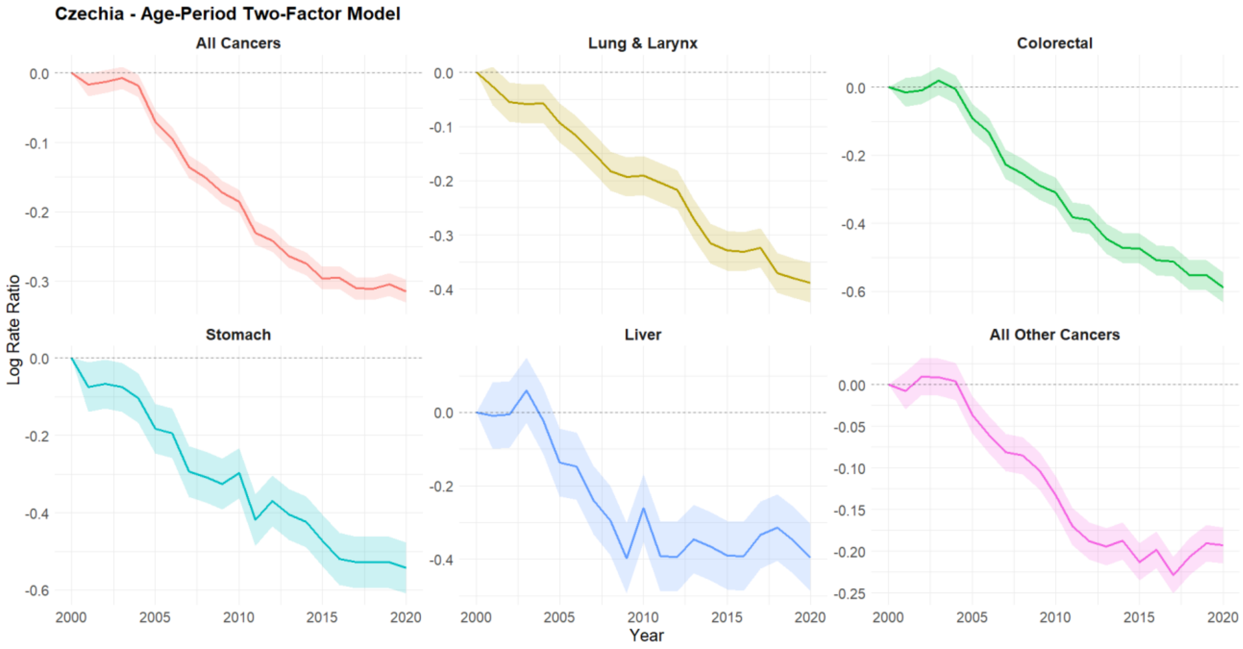

Figure A22: Log-linear cancer disease mortality rates for two-factor Age–Period models by year and type, Czechia, 2000–2020.

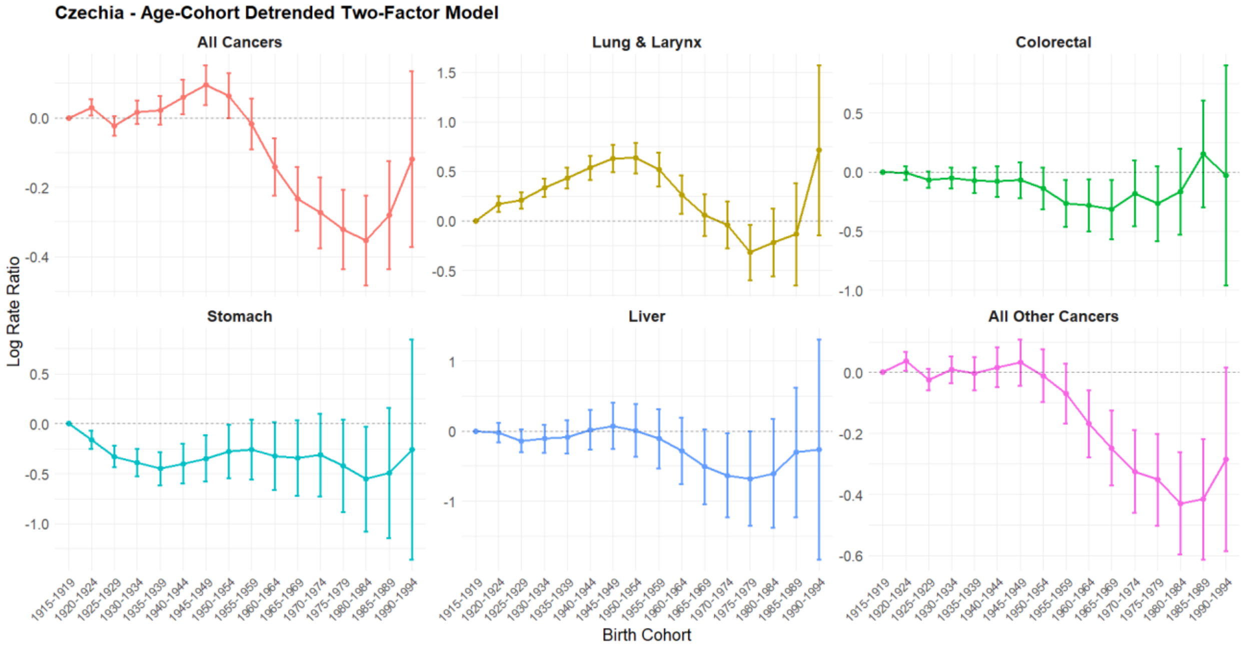

Figure A23: Log-linear cancer disease mortality rates for two-factor Age–Cohort models by year and type, Czechia, 2000–2020.

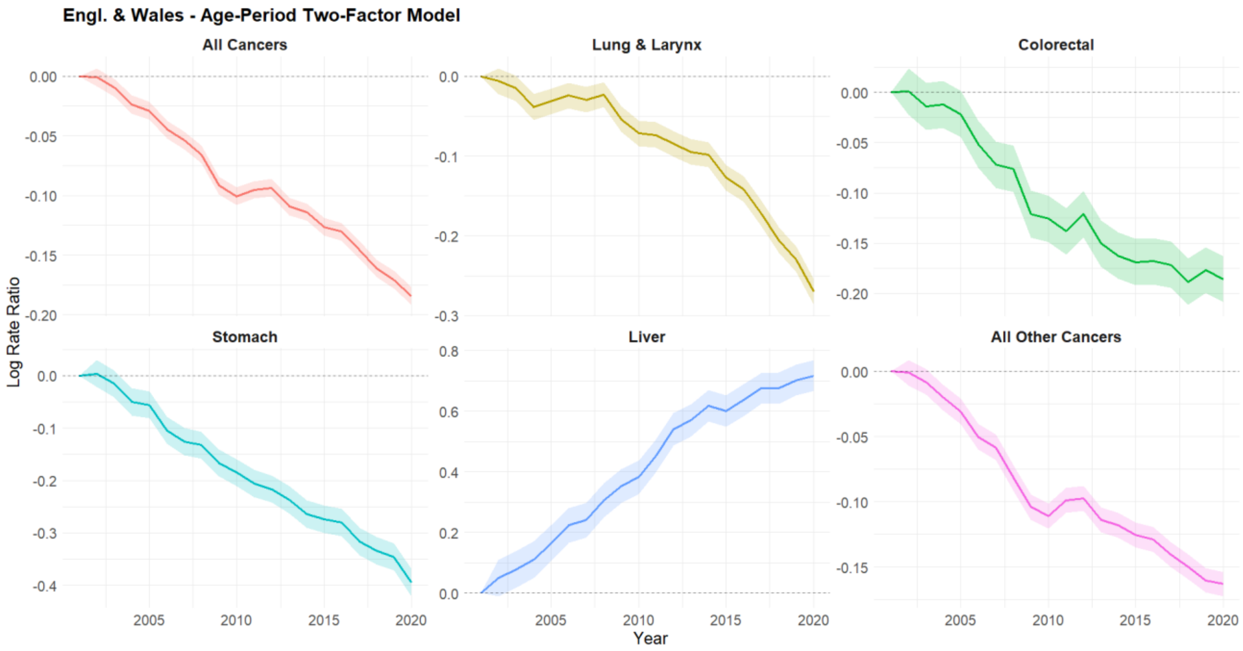

Figure A24: Log-linear cancer disease mortality rates for two-factor Age–Period models by year and type, England and Wales, 2001–2020.

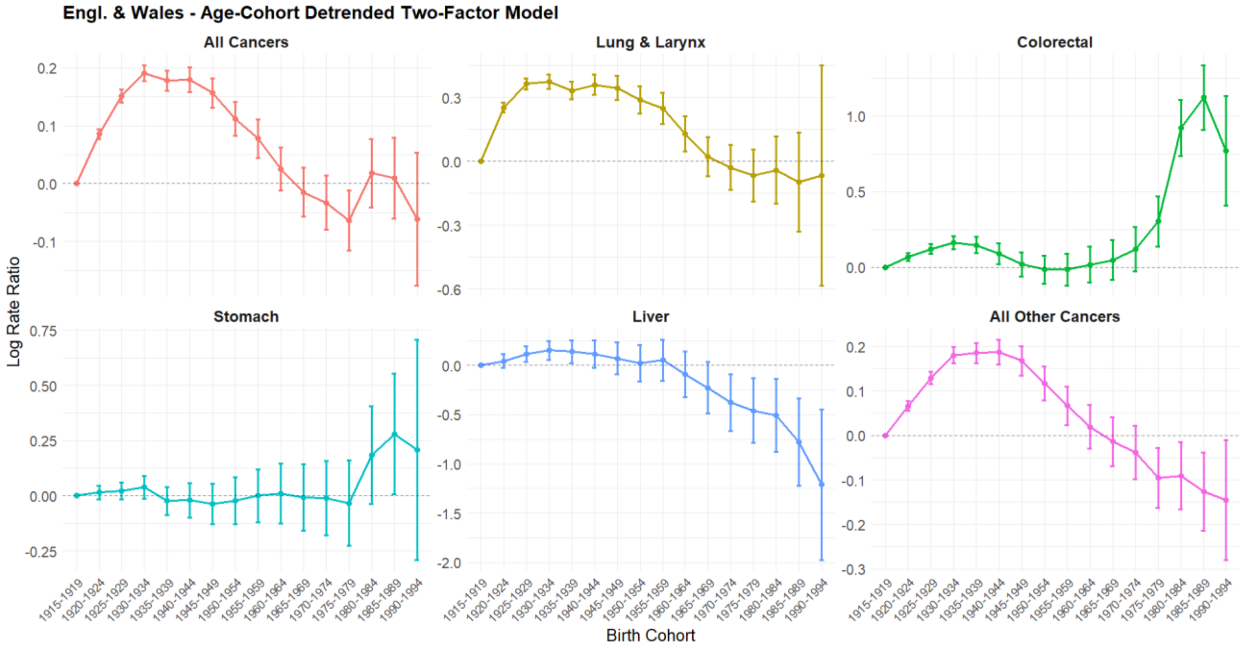

Figure A25: Log-linear cancer disease mortality rates for two-factor Age–Cohort models by year and type, England and Wales, 2001–2020.

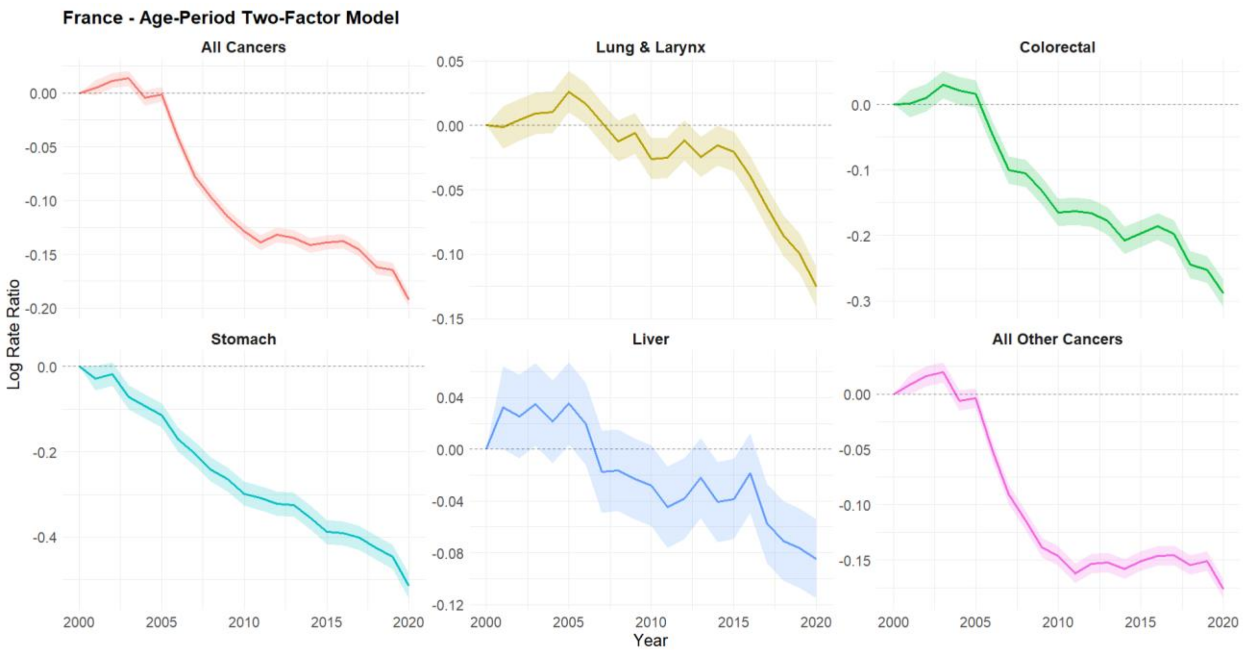

Figure A26: Log-linear cancer disease mortality rates for two-factor Age–Period models by year and type, France, 2000–2020.

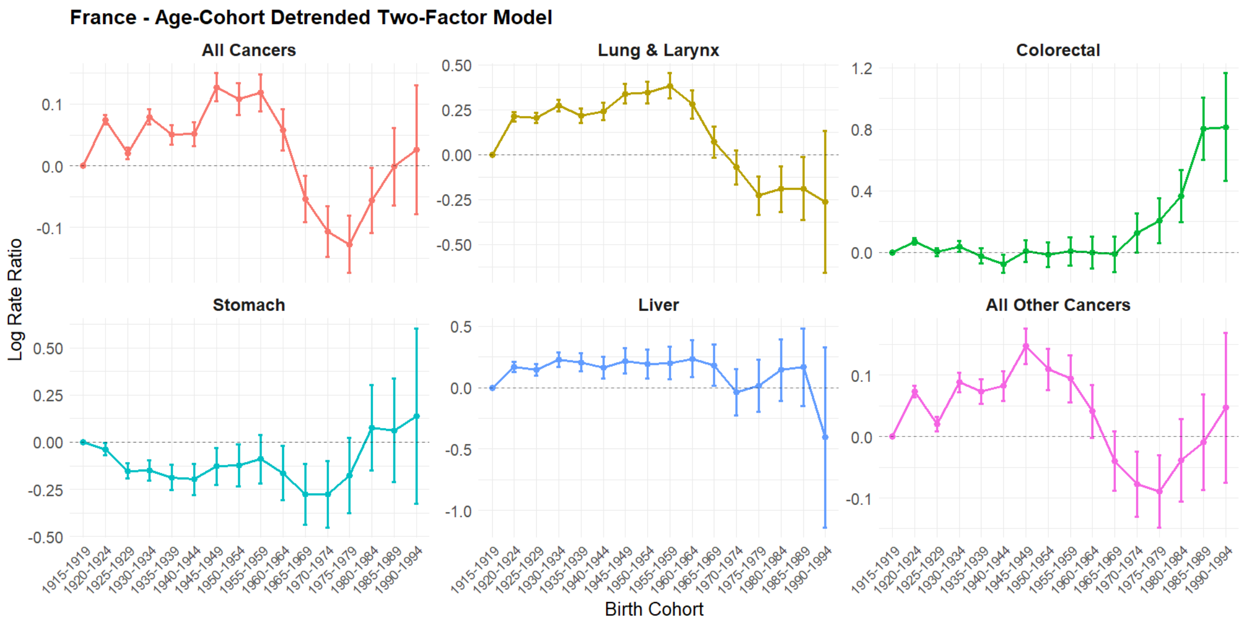

Figure A27: Log-linear cancer disease mortality rates for two-factor Age–Cohort models by year and type, France, 2000–2020.

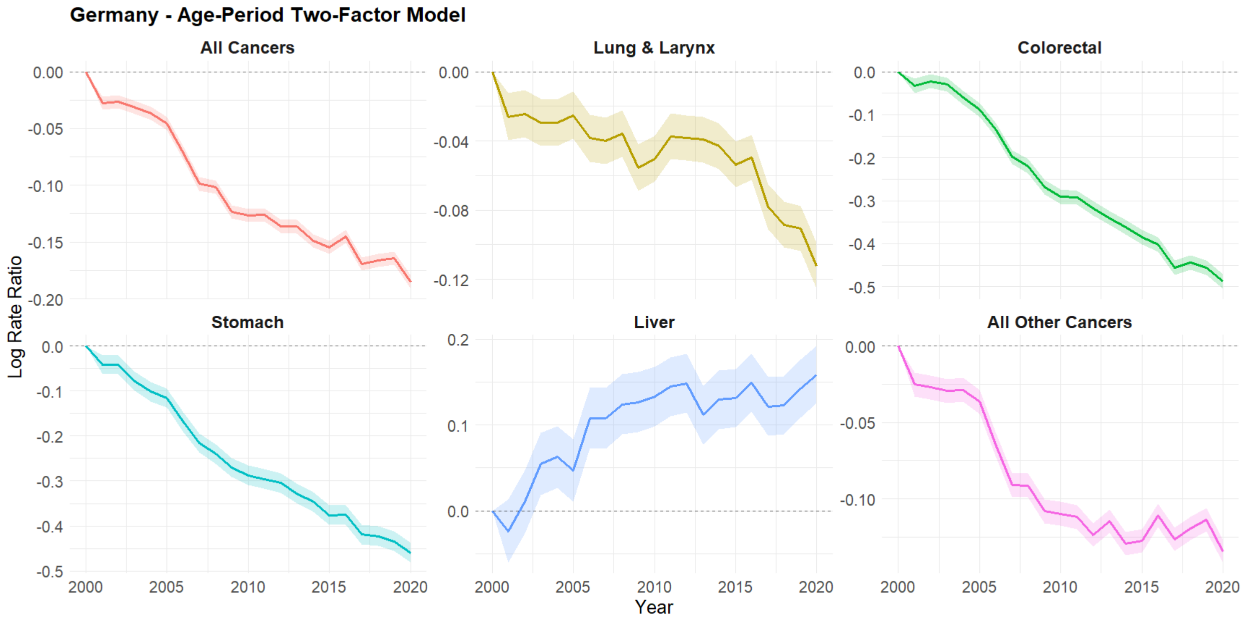

Figure A28: Log-linear cancer disease mortality rates for two-factor Age–Period models by year and type, Germany, 2000–2020.

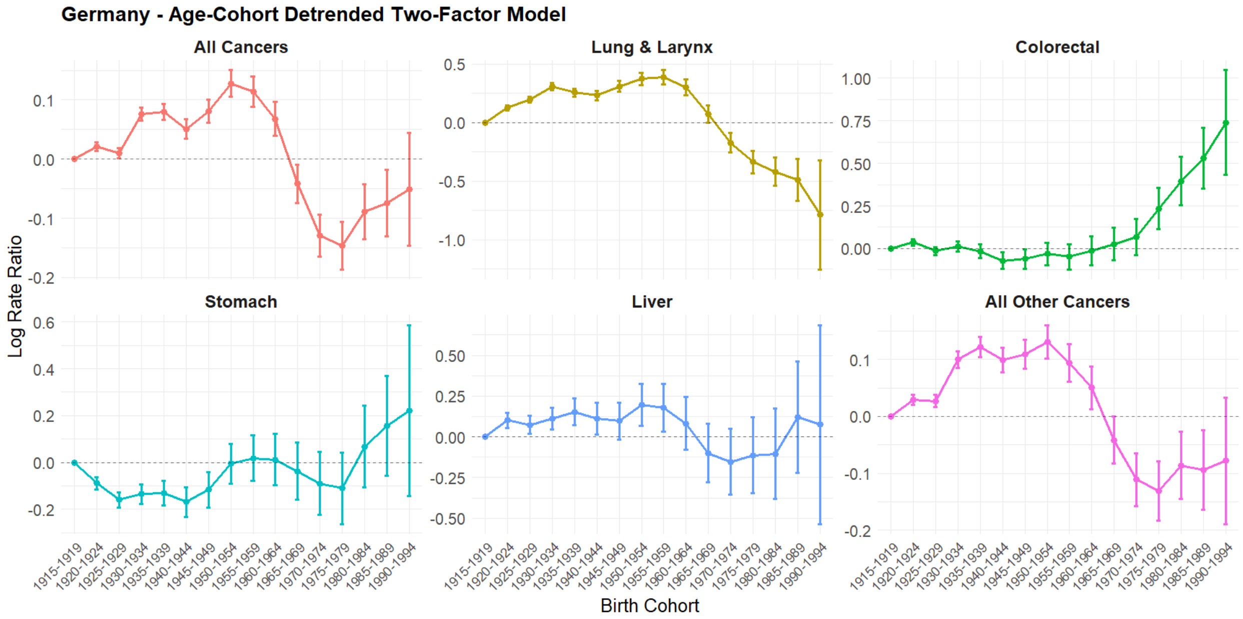

Figure A29: Log-linear cancer disease mortality rates for two-factor Age–Cohort models by year and type, Germany, 2000–2020.

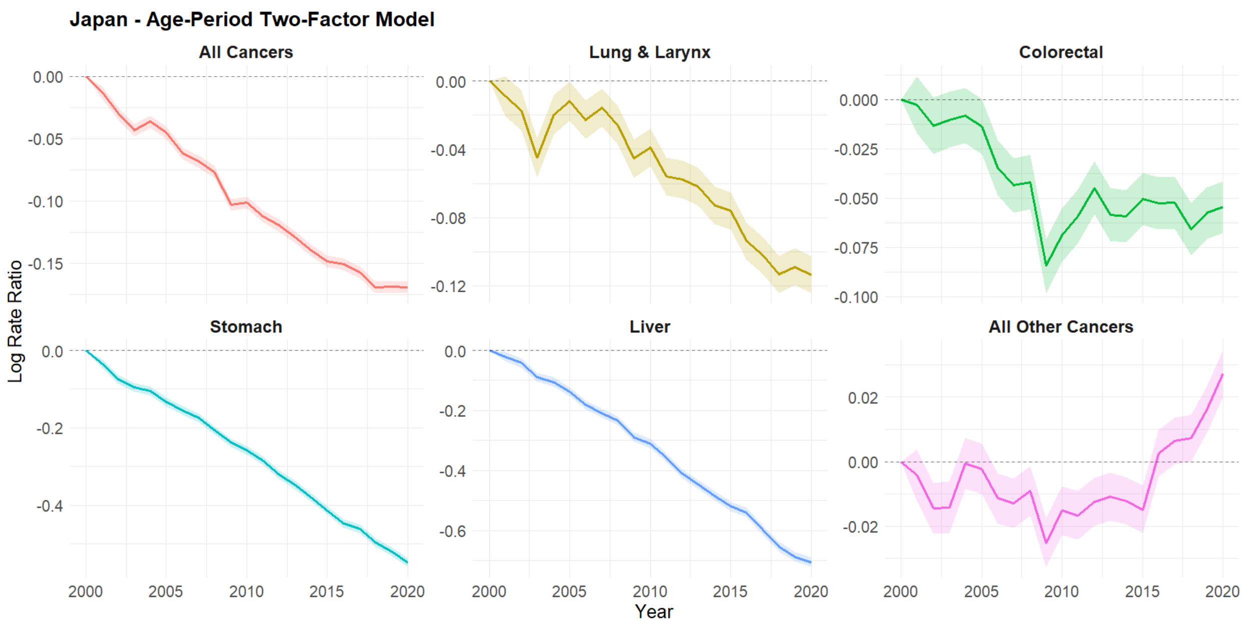

Figure A30: Log-linear cancer disease mortality rates for two-factor Age–Period models by year and type, Japan, 2000–2020.

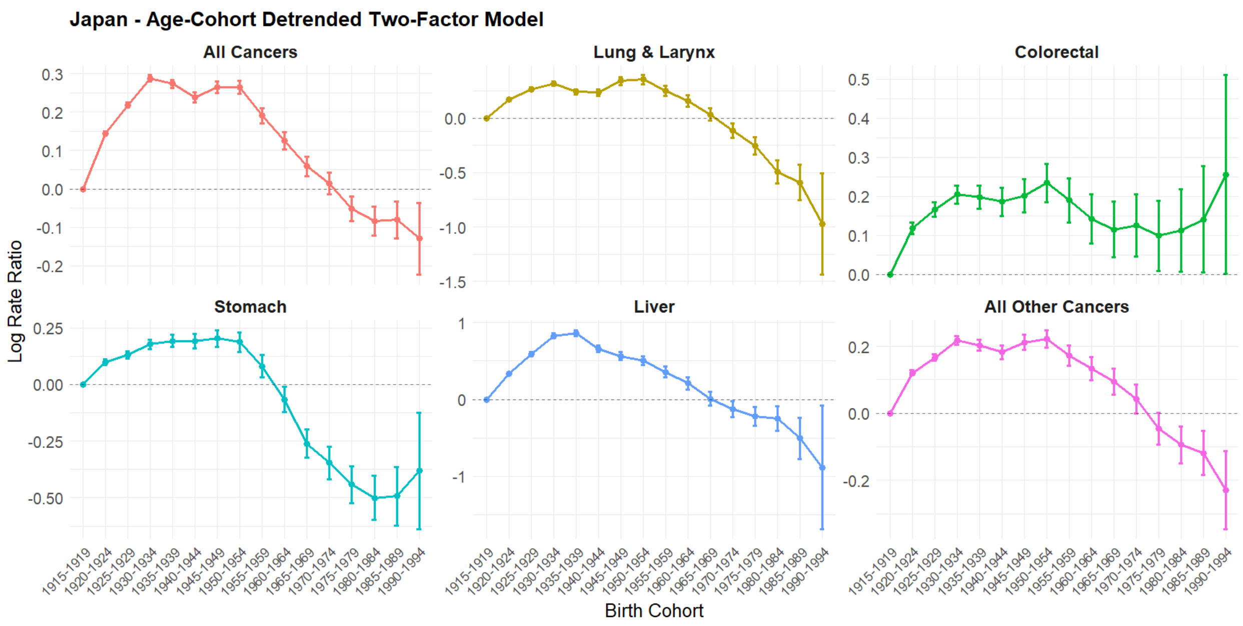

Figure A31: Log-linear cancer disease mortality rates for two-factor Age–Cohort models by year and type, Japan, 2000–2020.

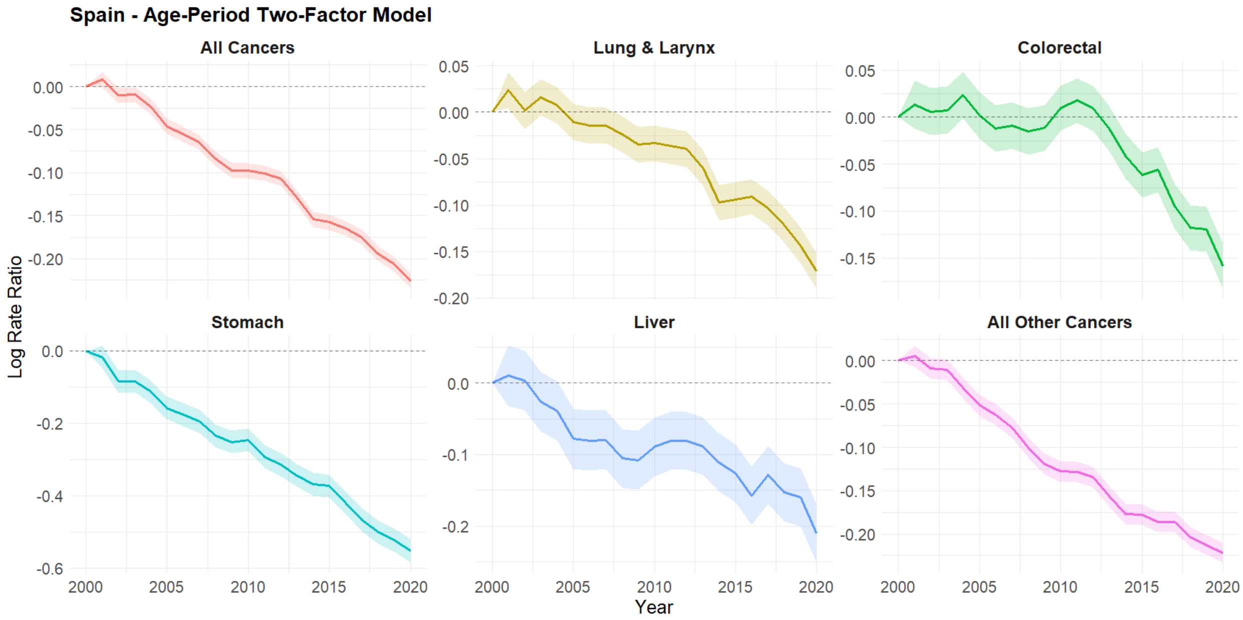

Figure A32: Log-linear cancer disease mortality rates for two-factor Age–Period models by year and type, Spain, 2000–2020.

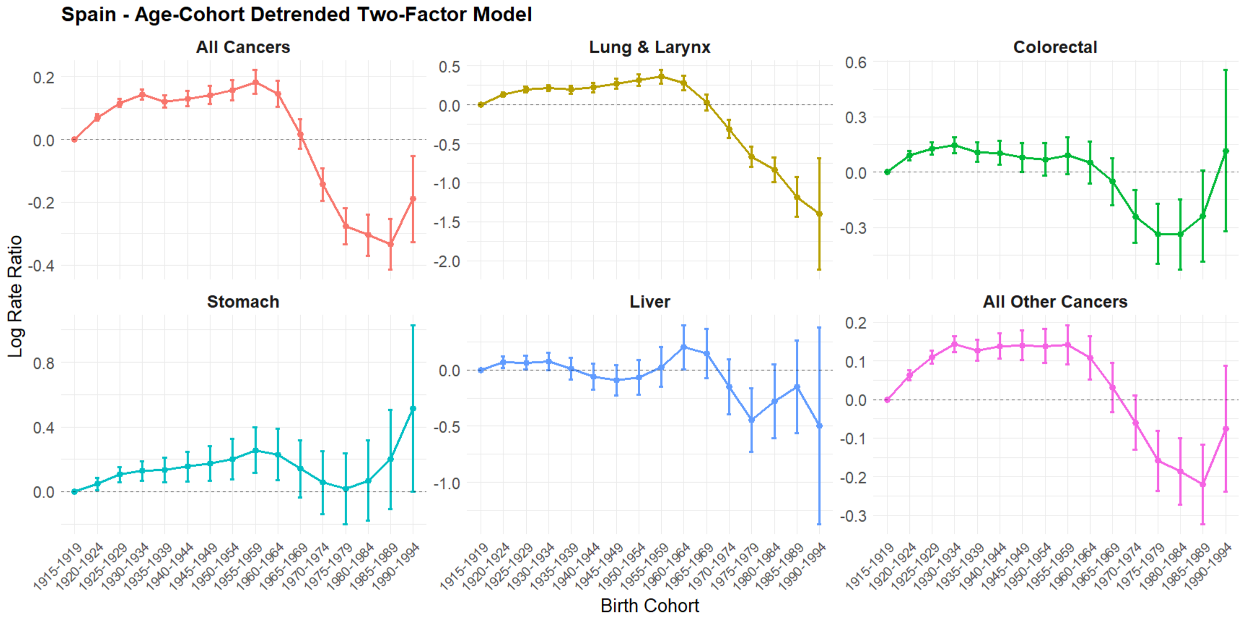

Figure A33: Log-linear cancer disease mortality rates for two-factor Age–Cohort models by year and type, Spain, 2000–2020.

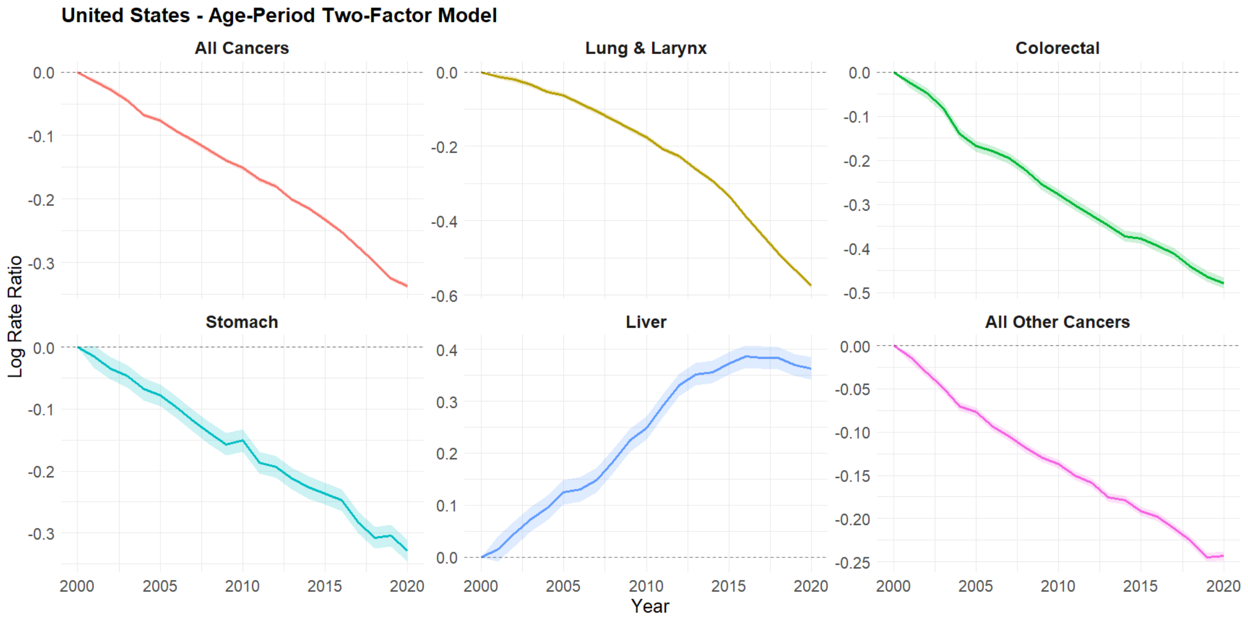

Figure A34: Log-linear cancer disease mortality rates for two-factor Age–Period models by year and type, United States, 2000–2020.

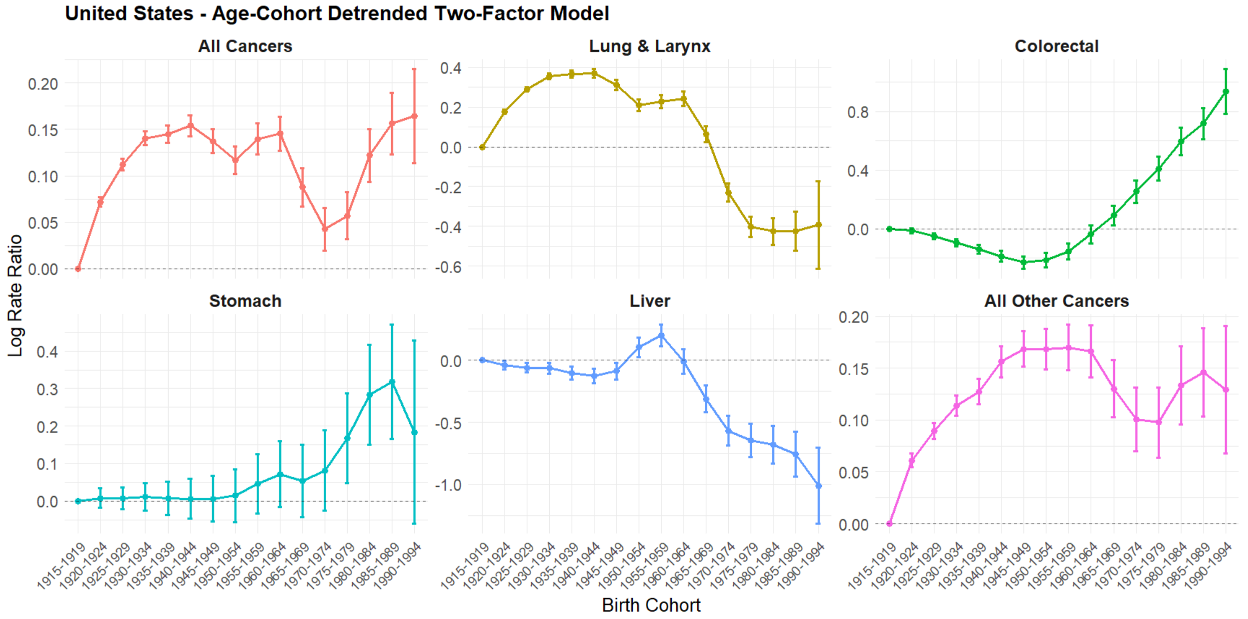

Figure A35: Log-linear cancer disease mortality rates for two-factor Age–Cohort models by year and type, United States, 2000–2020.

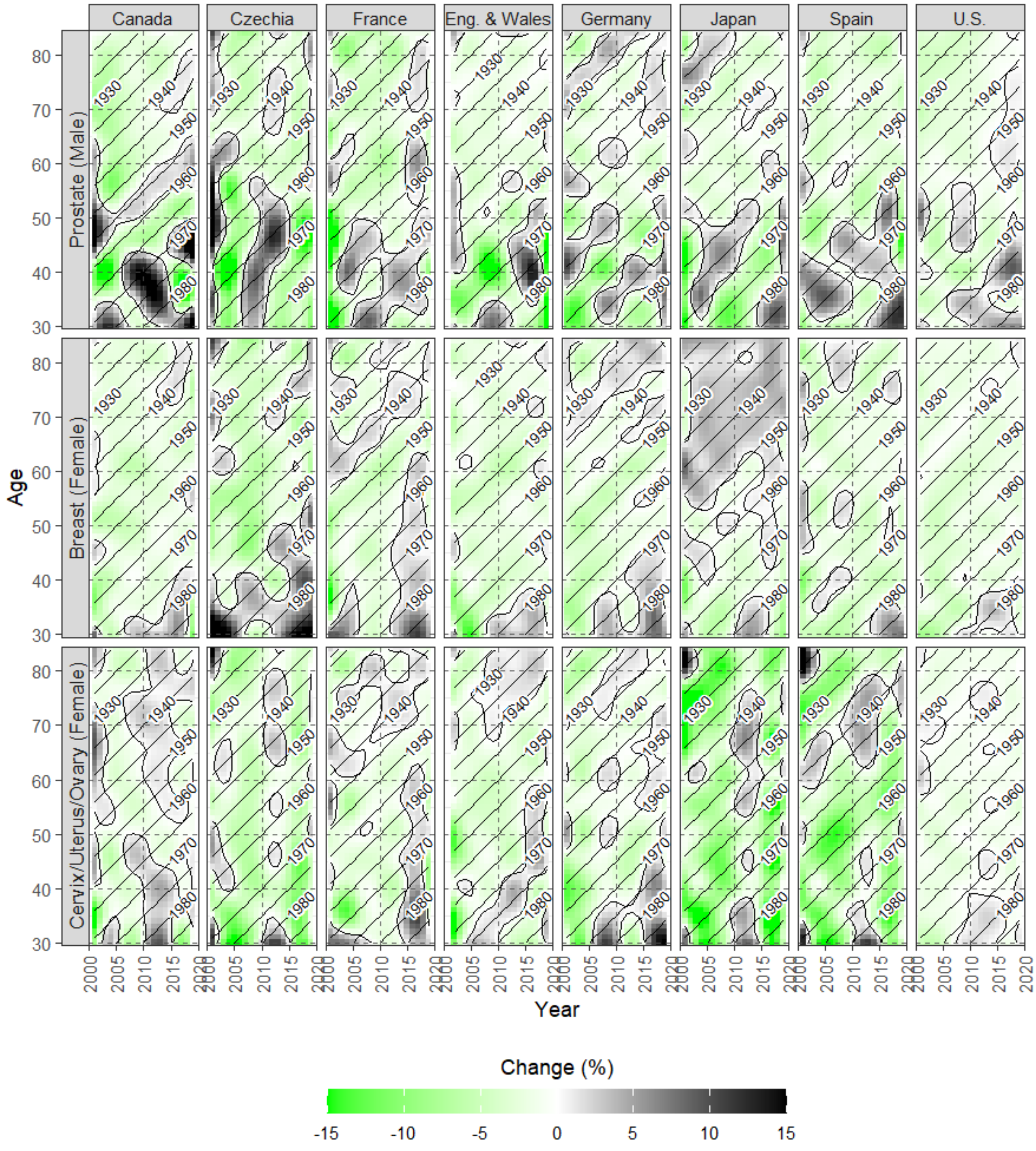

Figure A36: Rates of Mortality Improvement (%) across eight countries between 2000–2020, Reproductive cancers. Data from Human Cause of Death Data Series

Supplemental Appendix Table A1. ICD-10 and HCDs codes in each cause of death group

**Cause of death group**	**Individual causes included (and HCDs codes)**
Cancer	
Lung and larynx cancer	Malignant neoplasm of larynx C32; Malignant neoplasm of trachea, bronchus and lung C33, C34 **(L 056-057)**
Colorectal cancer	Malignant neoplasm of small intestine C17; Malignant neoplasm of colon C18; Malignant neoplasm of rectum, rectosigmoid junction and anus C19 (L051-052)
Stomach cancer	Malignant neoplasm of stomach C16 (L049)
Liver cancer	Malignant neoplasm of liver and intrahepatic bile ducts C22 (L053)
All other cancers	Malignant neoplasm of lip, oral cavity and pharynx C00-C14; Malignant neoplasm of esophagus C15; Malignant neoplasm of stomach C16; Malignant neoplasm of liver and intrahepatic bile ducts C22; Malignant neoplasm of pancreas C25; Malignant neoplasm of other and ill-defined digestive organs C23, C24, C26; Malignant neoplasm of other and ill-defined sites of respiratory and intrathoracic organs C30, C31, C37-C39, L059; Malignant neoplasm of bone and articular cartilage of limbs and other and unspecified sites C40, C41; Malignant melanoma of skin C43; Other malignant neoplasms of skin C44; Malignant neoplasm of mesothelioma and soft tissue C45-C49; Malignant neoplasm of breast C50; Malignant neoplasm of cervix uteri C53; Malignant neoplasm of corpus uteri and of uterus, part unspecified C54, C55; Malignant neoplasm of ovary C56; Malignant neoplasm of other and unspecified female genital organs C51, C52, C57, C58;

Malignant neoplasm of prostate C61; Malignant neoplasm of other male genital organs C60, C62, C63; Malignant neoplasm of kidney, except renal pelvis C64; Malignant neoplasm of bladder C67; Malignant neoplasm of other and unspecified urinary organs C65, C66, C68; Malignant neoplasm of meninges, brain, spinal cord, cranial nerves and other parts of central nervous system C70-C72; Malignant neoplasms of ill-defined, secondary and unspecified sites C69, C73-C80; Hodgkin's disease C81; Non-Hodgkin's lymphoma C82-C86; Multiple myeloma and malignant plasma cell neoplasms C90; Leukaemia C91-C95; Other and unspecified malignant neoplasms of lymphoid, haematopoietic, related tissue and malignant immunoproliferative diseases C88, C96
